# Functional and metabolic alterations of gut microbiota in children with new-onset type 1 diabetes

**DOI:** 10.1038/s41467-022-33656-4

**Published:** 2022-10-26

**Authors:** Xiaoxiao Yuan, Ruirui Wang, Bing Han, ChengJun Sun, Ruimin Chen, Haiyan Wei, Linqi Chen, Hongwei Du, Guimei Li, Yu Yang, Xiaojuan Chen, Lanwei Cui, Zhenran Xu, Junfen Fu, Jin Wu, Wei Gu, Zhihong Chen, Xin Fang, Hongxiu Yang, Zhe Su, Jing Wu, Qiuyue Li, Miaoying Zhang, Yufeng Zhou, Lei Zhang, Guang Ji, Feihong Luo

**Affiliations:** 1 Department of Pediatric Endocrinology and Inherited Metabolic Diseases, Children’s Hospital of Fudan University, Shanghai, China; 2 Shanghai Innovation Center of TCM Health Service, Shanghai University of Traditional Chinese Medicine, Shanghai, China; 3 Institute of Pediatrics, Children’s Hospital of Fudan University, Shanghai, China; 4 Shanghai Key Laboratory of Medical Epigenetics, Institutes of Biomedical Sciences, Fudan University, Shanghai, China; 5 Fuzhou Children’s Hospital of Fujian Medical University, Fuzhou, China; 6 Department of Endocrinology and Inherited Metabolic, Children’s Hospital Affiliated to Zhengzhou University, Zhengzhou, China; 7 Children’s Hospital of Soochow University, Suzhou, China; 8 The First Hospital of Jilin University, Changchun, China; 9 Department of Pediatric Endocrinology, Shandong Provincial Hospital Affiliated to Shandong First Medical University, Jinan, China; 10 The Affiliated Children’s Hospital of Nanchang University, Nanchang, China; 11 Department of Endocrinology, Genetics and Metabolism, The Children’s Hospital of Shanxi Province, Taiyuan, China; 12 The First Affiliated Hospital of Harbin Medical University, Harbin, China; 13 Department of Endocrinology, Children’s Hospital, Zhejiang University School of Medicine, Hangzhou, China; 14 Department of Pediatrics, West China Second University Hospital, Sichuan University, Chengdu, China; 15 Department of Endocrinology, Children’s Hospital of Nanjing Medical University, Nanjing, China; 16 Department of Neuroendocrinology Pediatrics, Affiliated Hospital of Qingdao University, Qingdao, China; 17 Fujian Medical University Union Hospital, Fuzhou, China; 18 Qingdao Women and Children’s Hospital, Qingdao, China; 19 Shenzhen Children’s Hospital, Shenzhen, China; 20 Institute of Pediatrics, Children’s Hospital of Fudan University, and the Shanghai Key Laboratory of Medical Epigenetics, International Co-laboratory of Medical Epigenetics and Metabolism, Ministry of Science and Technology, Institutes of Biomedical Sciences, Fudan University, Shanghai, China; 21 National Health Commission (NHC) Key Laboratory of Neonatal Diseases, Fudan University, Shanghai, China; 22 Institute of Digestive Diseases, Longhua Hospital, Shanghai University of Traditional Chinese Medicine, Shanghai, China

**Keywords:** Type 1 diabetes, Metagenomics

## Abstract

Gut dysbiosis has been linked to type 1 diabetes (T1D); however, microbial capacity in T1D remains unclear. Here, we integratively profiled gut microbial functional and metabolic alterations in children with new-onset T1D in independent cohorts and investigated the underlying mechanisms. In T1D, the microbiota was characterized by decreased butyrate production and bile acid metabolism and increased lipopolysaccharide biosynthesis at the species, gene, and metabolite levels. The combination of 18 bacterial species and fecal metabolites provided excellently discriminatory power for T1D. Gut microbiota from children with T1D induced elevated fasting glucose levels and declined insulin sensitivity in antibiotic-treated mice. In streptozotocin-induced T1D mice, butyrate and lipopolysaccharide exerted protective and destructive effects on islet structure and function, respectively. Lipopolysaccharide aggravated the pancreatic inflammatory response, while butyrate activated *Insulin1* and *Insulin2* gene expression. Our study revealed perturbed microbial functional and metabolic traits in T1D, providing potential avenues for microbiome-based prevention and intervention for T1D.

## Abstract (web summary)

Here, by performing multi-omics microbiome analyses in a cohort of children with new-onset T1D, the authors characterize altered microbial functional and metabolic traits involved in T1D, which they validate in animal experiments, together providing potential avenues for microbiome-based interventions.

## Introduction

Type 1 diabetes (T1D) is characterized by beta-cell destruction and insulin deficiency induced by autoimmune attacks associated with genetic predisposition^1^. However, the incidence of T1D has been steadily increasing annually worldwide over the past 30 years^2^, and even two times higher in rapidly developing regions of China^3,4^, highlighting the importance of environmental shifts in the pathogenesis of T1D^5^. The etiology of T1D is multifactorial, consisting of genetic susceptibility and environmental factors including viral infections, dietary components, and gut microbiota alterations^6^.

Clinically, patients with T1D have been widely reported with “leaky gut,” described as increased gut permeability^7–9^. Structural dysbiosis of the gut microbiota in T1D has been reported, as indicated by the decreased diversity^9^ and Firmicutes/Bacteroidetes ratio^10^, and the absence of short-chain fatty acid (SCFA) producers^11,12^. Notably, a study on the environmental determinants of diabetes in the young cohort (TEDDY) revealed taxonomically diffuse but coherent functional microbial signatures across geographically diverse centers, characterized by a decreased abundance of microbial genes related to fermentation and SCFA biosynthesis^13^. Integrated metagenomics and metaproteomics analyses have revealed the associations of structural and functional microbial signatures with host proteomic traits in the T1D genealogical^14^ and new-onset cohort^7^. A recent study showed the alteration of gut microbiome composition in patients with long-standing T1D and its association with glycemic control and complications^15^.

Studies in animal models also showed that gut microbiota may play a causal role in the onset and progression of T1D. Exposure of congenic mice to the normal gut microbiota attenuates the process of T1D in germ-free MyD88-deficient mice with T1D^16^. Further, maternal cecal microbiota could rescue antibiotic-induced T1D enhancement in non-obese diabetic (NOD) mice^17^. Furthermore, the impairment of gut barrier integrity triggers the activation of islet-specific T cells and autoimmune diabetes, which depends on the presence of gut microbiota^18^.

However, the comprehensive profiling of functional and metabolic dysbiosis of gut microbiota in T1D is lacking, and the microbial signatures associated with T1D pathogenesis require further identification. Here, to decipher the T1D-associated functional features of gut dysbiosis, we performed integrative metagenomic and metabolomic analyses in the independent discovery and validation cohort of pediatric patients with new-onset T1D and identified T1D-associated microbial species, functional pathways, and fecal metabolites that might be related to T1D risk. We also performed fecal microbiota transplantation (FMT) and established the T1D mouse model to elucidate the mechanisms whereby the gut microbiota and its metabolites interact with the host in the progression of T1D.

## Results

### Anthropometric and biochemical measurements of T1D

Through a strict pathological diagnostic and exclusion process, children with new-onset T1D and non-diabetic control (NC) subjects were enrolled in the discovery and validation cohorts, respectively (Supplementary Fig. 1 and 2). The two groups were matched for age, sex, delivery, and feeding mode. With a lower body mass index (BMI), the T1D group showed poor glycemic control and low C-peptide levels. The levels of systemic inflammatory parameters, including white blood cell (WBC) count, and neutrophil (NEUT) number, were significantly increased in the T1D group compared with those in the NC group. In addition, the T1D group showed a significant decrease in high-density lipoprotein cholesterol (HDL-C) levels and an increase in triglyceride (TG) levels compared with the NC group. Similar findings were obtained in the validation cohort (Table 1).

**Table 1:** Characteristics of study participants

Parameter	Discovery set (*n* = 141)			Validation set (*n* = 58)		
	NC group (*n* = 77)	T1D group (*n* = 64)	*P* value	NC group (*n* = 29)	T1D group (*n* = 29)	*P* value
Female, *n* (%)	32 (41.6)	26 (40.6)	0.911	13 (44.8)	14 (48.3)	0.792
Age (years)	7.92 ± 2.92	7.53 ± 3.61	0.486	6.48 ± 2.21	6.58 ± 1.96	0.848
BMI (kg/m^2^)	15.81 ± 1.81	14.81 ± 2.26	0.005	16.12 ± 3.05	14.32 ± 1.61	0.014
BMI *z* score	−0.17 ± 0.97	−1.11 ± 1.55	<0.001	0.11 ± 1.38	−0.98 ± 0.99	0.002
HbA1c (%)	5.06 ± 0.51	11.99 ± 2.46	<0.001	4.88 ± 0.41	12.16 ± 2.31	<0.001
FBG (mmol/L)	4.91 (4.74, 5.12)	17.20 (10.40, 24.69)	<0.001	4.80 (4.02, 4.94)	15.13 (9.85, 29.15)	<0.001
C-peptide (ng/mL)	ND	0.195 (0.10, 0.40)		ND	0.33 (0.17, 0.45)	
WBC (×10^9^/L)	6.70 (5.69, 8.13)	7.79 (5.43, 10.53)	0.032	6.82 (6.05, 8.20)	7.40 (6.38, 9.63)	0.195
NEUT (×10^9^/L)	3.15 (2.56, 4.29)	4.03 (2.43, 6.32)	0.049	3.25 (2.69, 4.01)	3.70 (2.88, 5.43)	0.086
LYMPH (×10^9^/L)	2.88 (2.18, 3.31)	2.95 (2.32, 3.68)	0.266	3.00 (2.47, 3.84)	2.85 (2.40, 4.16)	0.960
Cholesterol (mmol/L)	4.27 (3.70, 4.86)	4.46 (3.70, 5.70)	0.147	3.77 (3.55, 4.61)	4.09 (3.79, 5.61)	0.085
Triglycerides (mmol/L)	0.65 (0.51, 0.88)	1.31 (0.78, 3.38)	<0.001	0.54 (0.47, 0.74)	1.21 (0.74, 2.11)	<0.001
HDL-C (mmol/L)	1.43 (1.27, 1.74)	1.22 (1.03, 1.45)	<0.001	1.48 (1.29, 1.78)	1.05 (0.94, 1.43)	0.002
LDL-C (mmol/L)	2.45 (2.10, 2.93)	2.74 (2.07, 3.27)	0.242	2.04 (1.87, 2.31)	2.30 (1.95, 2.69)	0.121
Vaginal delivery, *n* (%)	39 (52.0)	36 (56.3)	0.616	11 (40.7)	12 (41.4)	0.961
Breastfeeding time (months)	6.0 (2.0, 10.0)	6.0 (5.0, 10.0)	0.422	10.5 (5.0, 12.8)	6.0 (2.0, 12.0)	0.370
Complementary feeding (months)	6.0 (5.4, 6.1)	6.0 (5.0, 6.0)	0.142	6.0 (4.0, 6.0)	6.0 (5.0, 6.0)	0.502

Data are presented as mean ± standard deviation (SD), median with interquartile range (IQR), or *n* (%). The *p* values are based on the two-sided *t* test for variables expressed as mean ± SD, Wilcoxon rank-sum test for variables expressed as median (IQR) and chi-square Test for variables expressed as percentages. NC vs T1D group. *NC* normal control, *T1D* type 1 diabetes, *ND* not determined, *BMI* body mass index, *HbA1c* hemoglobin A1c, *FBG* fasting blood glucose, *WBC* white blood cell, *NEUT* neutrophil, *LYMPH* lymphocytes, *HDL-C* high-density lipoprotein, *LDL-C* low-density lipoprotein.

### Structural modulation of the gut microbiota in T1D

**Fig. 1:**
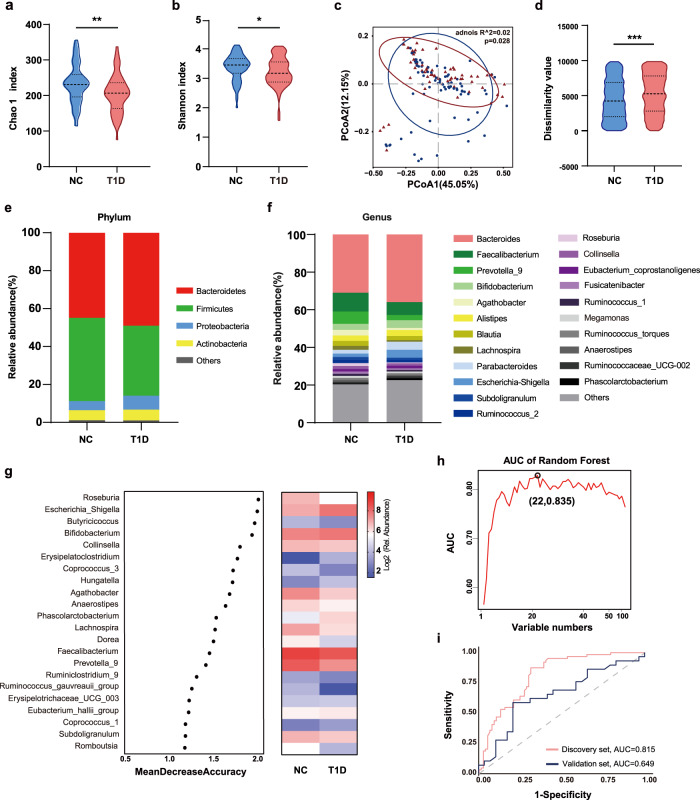
The structural shift of gut microbiota in children with T1D. **a**, **b** The microbial community richness (Chao 1 index; **a**, *p* = 0.003) and diversity (Shannon index; **b**, *p* = 0.035). **c**, **d** Principal coordinate analysis (PCoA) analysis based on weighted UniFrac distance (**c**) and inner-group distance by analysis of similarities (ANOSIM) (**d**, *p* < 0.001). **e**, **f** The relative abundance of microbial taxa at phylum and genus levels; phyla or genera with a relative abundance <1% in each sample are merged into others. **g** Classification performance of the 22 most discriminant genera of the T1D group by a random forest model and heatmap based on the relative abundance of the genera. **h** Area under the curve (AUC) based on the cross-validation of the random forest model in the discovery set. **i** Receiver operating characteristic (ROC) curves and their corresponding AUCs employing 22 genera in the discovery and validation sets. For the discovery set, NC: *n* = 77, T1D: *n* = 64. For the validation set, NC: *n* = 29, T1D: *n* = 29. Violin plots show the median, quartiles, and min/max values. Two-sided Wilcoxon rank-sum test. *p < 0.05, **p < 0.01, ***p < 0.001. Source data are provided as a Source Data file.

First, we performed high-throughput sequencing of the V3-V4 areas of the 16 S ribosomal RNA (rRNA) gene to profile the structure of gut microbiota in T1D. The Chao 1 and Shannon indices, which reflect the richness and diversity of the microbiota, were significantly lower in the T1D group than in the NC group (Fig. 1a, b). Principal coordinate analysis (PCoA) based on weighted UniFrac distance metrics showed a significant difference in the microbial community structure between the two groups (permutational multivariate analysis of variance [PERMANOVA]: *p* = 0.028; R-squared = 0.02). We also observed increased compositional dissimilarity in the T1D group, indicating a more heterogeneous community structure among T1D individuals than in controls (Fig. 1c, d). At the phylum level, the T1D group was characterized by a significantly lower abundance of Firmicutes and higher abundance of Bacteroidetes and Proteobacteria than in the NC group (Fig. 1e). At the genus level (Fig. 1f and Supplementary Fig. 3), dysbiosis in T1D was characterized by a decrease in many butyrate-producing genera within Firmicutes, including *Faecalibacterium, Blautia, Lachnospira, Ruminococcus 2*, and *Roseburia*, and an expansion of genera such as *Bacteroides, Parabacteroides*, and *Escherichia Shigella*. A similar microbiota profile of T1D was detected in the validation group (Supplementary Fig. 4). Moreover, we performed subgroup analysis according to geographical regions and disease status (experienced diabetic ketoacidosis (DKA) or not), and no significant differences were found in overall bacterial structures both in the discovery and validation set (Supplementary Figs. 5 and 6).

Using random forest analysis, we constructed the optimal classifier model of T1D using 126 highly abundant genera (Fig. 1g, h, and Supplementary Data 1). A group of 22 genera was selected as the key genera that provided the best discriminatory power by leave-one-out cross-validation. These key genera showed a moderate distinguishing effect on T1D, with area under the curve (AUC) values of 0.815 and 0.649 in the discovery and validation sets, respectively (Fig. 1i).

**Fig. 2:**
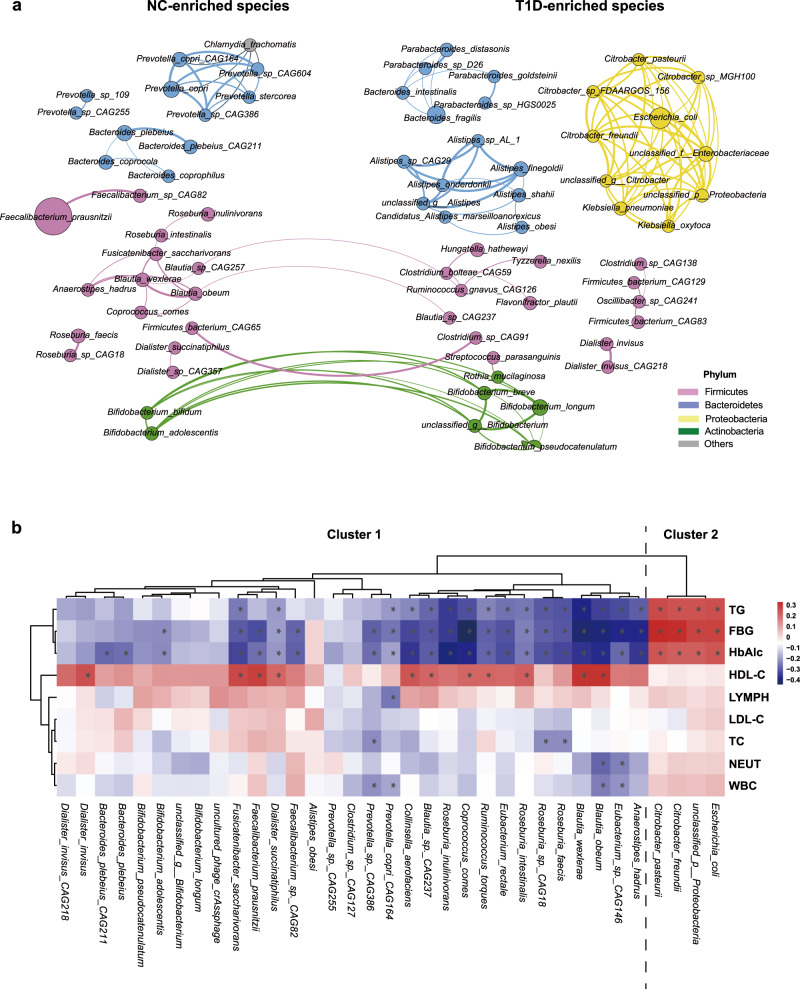
The T1D-associated microbial species revealed by metagenomic analysis. **a** Co-abundance network constructed from the microbial markers of T1D by a random forest model using the top 100 most abundant species. The node size indicates the relative abundance of each bacteria species and color indicates the phylum. The thickness of the line between nodes represents the Spearman coefficient. A total of 72 species were displayed with Spearman’s correlation values over 0.6 between each other. NC-enriched species are arranged on the left, while T1D-enriched species are arranged on the right. **b** Heatmap of the Spearman’s correlation between clinical indices and discriminatory species (*FDR < 0.05). Red squares indicate positive correlations, whereas blue squares indicate negative correlations. NC: *n* = 77, T1D: *n* = 64. *FDR < 0.05. Source data are provided as a Source Data file.

The microbial community structure at the species level was further analyzed using metagenomic sequencing. As shown in the network analysis, the abundances of many butyrate-producing species, such as *Faecalibacterium prausnitzii*, *Eubacterium rectale*, and *Roseburia intestinalis*, were reduced in the T1D group, compared with those in the NC group. In contrast, many opportunistic pathogens, including *Escherichia coli, unclassified Enterobacteriaceae*, and *Klebsiella pneumoniae*, formed a close cluster and were enriched in the T1D group (Fig. 2a). Moreover, the network formed by the species in each group was more interconnected in the NC group than in the T1D group (Supplementary Fig. 7). Based on the random forest model, 35 species were selected as the most discriminatory species for T1D (Supplementary Fig. 8). These bacterial species were grouped into two clusters. Cluster 1 mainly consisted of butyrate-producing bacteria, whereas Cluster 2 mainly comprised opportunistic pathogens. Spearman’s correlation analysis between clinical indices and discriminatory species revealed that hemoglobin A1c (HbA1c), fasting blood glucose (FBG), and TG levels were negatively correlated with the species in Cluster 1 and positively correlated with those in Cluster 2, whereas HDL-C showed the opposite pattern (Fig. 2b). Furthermore, indices representing systemic inflammation, such as WBC, NEUT, and lymphocyte (LYMPH) levels, were negatively correlated with many species in Cluster 1. The multiple correlations between metabolic parameters and key species suggest that the gut microbiota may be involved in the regulation of glucose metabolism, lipid metabolism, and inflammatory responses in T1D.

### Functional alterations of the gut microbiota in T1D

**Fig. 3:**
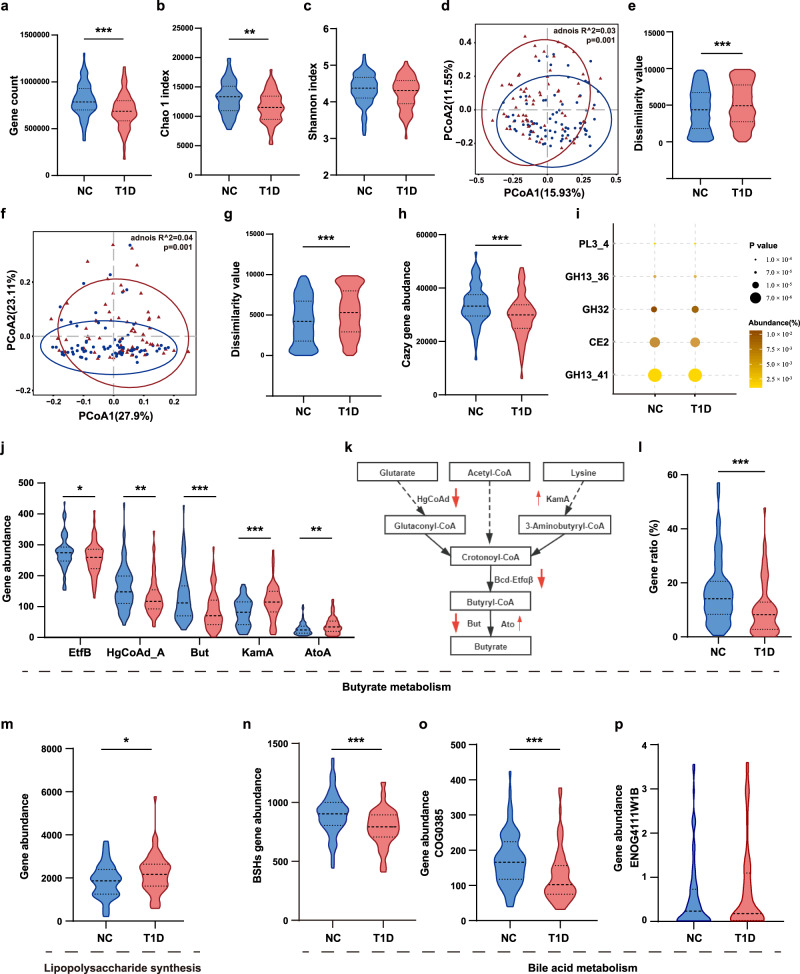
Alteration of microbial functions in children with T1D. **a** Total gene count in the NC and T1D groups (*p* < 0.001). **b**, **c** The microbial community richness (Chao 1 index; **b**, *p* = 0.002) and diversity (Shannon index; **c**). **d**, **e** PCoA based on weighted UniFrac distance (**d**) and inner-group distance by ANOSIM (**e**, *p* < 0.001). **f**, **g** PCoA plot based on the Bray-Curtis distances of KOs (**f**) and inner-group distance by ANOSIM (**g**, *p* < 0.001). **h** The abundance of carbohydrate-active enzymes (CAZy) genes (*p* < 0.001). **i** The five most differential expressed CAZy genes. **j** The abundance of the most differential expressed genes involved in butyrate metabolism (*EefB*, *p* = 0.044; *HgCoAd_A*, *p* = 0.002; *But*, *p* < 0.001; *KamA*, *p* < 0.001; *AtoA, p* = 0.009). **k** The metabolic pathways for butyrate synthesis. The upward and downward red arrows represent upregulation and downregulation in the T1D group compared to the NC group. **l** The contribution of *Faecalibacterium prausnitzii* to genes involved in butyrate metabolism (*p* < 0.001). **m** The abundance of genes involved in lipopolysaccharide synthesis based on the KEGG database (*p* = 0.019). **n** The abundance of genes involved in BSHs based on the UniProt database (*p* < 0.001). **o**, **p** The abundance of genes involved in bile acid metabolism based on the EggNOG database (COG0385*, p* < 0.001). NC: *n* = 77, T1D: *n* = 64. Violin plots show the median, quartiles, and min/max values. Two-sided Wilcoxon rank-sum test. *p < 0.05, **p < 0.01, ***p < 0.001. Source data are provided as a Source Data file.

To further identify the functional features of the gut microbiome in T1D, we performed the metagenomic sequencing of the fecal samples (Supplementary Data 2 and 3). The T1D group showed a decreased gene count (Fig. 3a) and Chao 1 (Fig. 3b) and Shannon (Fig. 3c) indices compared with the NC group. PCoA based on the weighted UniFrac distance of species also showed a significant difference between groups (PERMANOVA: *p* = 0.001; R-squared = 0.035), with a higher compositional dissimilarity in the T1D group than in case of the controls (Fig. 3d, e). A PCoA plot based on the Bray-Curtis distances of the Kyoto Encyclopedia of Genes and Genomes (KEGG) orthologs (KOs) indicated significant differences (PERMANOVA: *p* = 0.001; R-squared = 0.036) between the two groups at the functional level (Fig. 3f, g and Supplementary Data 4). Using linear discriminant analysis effect size, we detected 27 upregulated and 29 downregulated pathways in the T1D group compared with those in the controls that were mainly involved in carbohydrate, amino acid, and nucleotide metabolism (Supplementary Fig. 9). Notably, the T1D group showed lower levels of starch and sucrose metabolism, and increased lipopolysaccharide (LPS) synthesis than the NC group.

Furthermore, the total abundance of genes encoding carbohydrate-active enzymes (CAZy) was significantly lower in the T1D group than in the NC group (Fig. 3h, i, and Supplementary Fig. 10). Notably, the most differentially expressed CAZy genes, which were mainly related to starch, glycogen, sucrose, and fructose metabolism, were all decreased in the T1D group compared with that in the NC group (Supplementary Data 5). With a focus on butyrate metabolism, we further performed alignment based on a database^19^ concerning butyrate-producing pathways and found that most genes involved in the butyrate production pathway were downregulated in the T1D group compared with that in the controls (Fig. 3j, k, and Supplementary Data 6). We then constructed the draft genome of *Faecalibacterium prausnitzii* (Supplementary Fig. 11) and found that this species contributed up to 13.58% of the total abundance of genes related to butyrate metabolism, whereas it accounted for 16.27% and 10.35% of the total abundance of genes in the NC and T1D groups, respectively (Fig. 3l and Supplementary Data 7). Moreover, the total abundance of genes related to LPS synthesis increased significantly in the T1D group compared with that in the NC group (Fig. 3m). We also found that the total abundance of genes encoding microbial bile salt hydrolases (BSHs) and bile acid metabolism decreased significantly in the T1D group compared with those in controls (Fig. 3n–p and Supplementary Data 8). Overall, the metagenomic analysis revealed a disturbed microbial functional profile in the T1D group, including increased LPS biosynthesis and decreased butyrate production, carbohydrate metabolism, and bile acid metabolism compared with those in controls.

### Aberrant metabolic activities in T1D

**Fig. 4:**
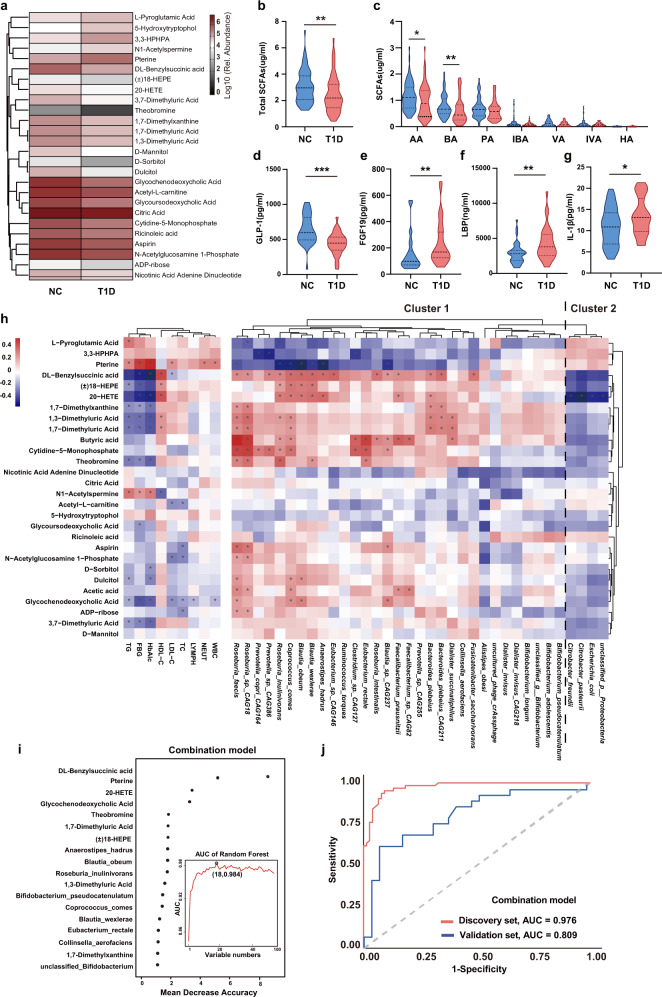
Aberrant fecal metabolic patterns in children with T1D. **a** Heatmap of the relative abundance of 26 significantly different metabolites between the NC and T1D group. Metabolites with VIP ≥ 1 and fold change ≥2 or fold change ≤0.5 were considered differential metabolites. **b**, **c** The concentration of total and seven fecal short-chain fatty acids, respectively (total SCFAs, *p* = 0.004; AA, *p* = 0.024; BA, *p* = 0.005). **d**–**g** The serum concentration of **d** GLP-1 (*p* < 0.001), **e** FGF19 (*p* = 0.002), **f** LBP (*p* = 0.003), and **g** IL-1β (*p* = 0.042) in the NC and T1D group (NC: *n* = 40, T1D: *n* = 34). **h** Heatmap of the Spearman’s correlation between 28 discriminatory metabolites and 35 key bacteria species as well as clinical parameters (*FDR < 0.05). The red squares indicate positive correlations, whereas the blue squares indicate negative correlations. **i** Metabolomic and metagenomic markers for detecting the T1D group from the NC group were identified from Random-forest classifiers based on the combination of dual-omics markers. Markers are ranked in descending order of their importance to the accuracy of the model. **j** Receiver operating characteristic (ROC) curves and their corresponding area under curve (AUC), employing the combination of dual-omics markers. AA acetic acid, BA butyric acid, PA propionic acid, IBA isobutyric acid, VA valeric acid, IVA isovaleric acid, HA hexanoic acid. GLP-1 glucagon-like peptide 1, FGF19 fibroblast growth factor 19, LBP lipopolysaccharide-binding protein. For metabonomic analysis, NC: *n* = 77, T1D: *n* = 64 in the discovery set, NC: *n* = 29, T1D: *n* = 29 in the validation set. Violin plots show the median, quartiles, and min/max values. Two-sided Wilcoxon rank-sum test. *p < 0.05, **p < 0.01, ***p < 0.001. Source data are provided as a Source Data file.

To further investigate the biological effects of the gut microbiota in T1D, metabolomic analysis was performed using fecal samples. Compared with those in the NC group, the levels of five metabolites (L-pyroglutamic acid, pterine, 5-hydroxytryptophol, N1-acetylspermine, and 3-(3-hydroxyphenyl)-3-hydroxypropanoic acid) increased significantly, while those of 21 metabolites, including glycoursodeoxycholic acid, glycochenodeoxycholic acid, and DL-benzylsuccinic acid, decreased significantly in the T1D group (Fig. 4a and Supplementary Fig. 12). These discriminant metabolites belonged to 11 metabolic pathways, mainly related to bile acid, carbohydrate, nucleotide, and amino acid metabolism (Supplementary Data 9). According to KEGG pathway enrichment analysis, three pathways were significantly enriched (Supplementary Fig. 12c): fructose and mannose, galactose, and caffeine metabolic pathways. We also found that the total concentrations of fecal SCFAs, butyrate, and acetic acid were significantly lower in the T1D group than in the NC group (Fig. 4b, c). Moreover, the serum concentration of glucagon-like peptide 1 (GLP-1), whose secretion could be stimulated by butyrate, was lower in the T1D group than in the NC group (Fig. 4d). The level of fibroblast growth factor 19 (FGF19), a negative feedback regulator of hepatic bile acid synthesis^20^, was significantly elevated in the T1D group compared with that in the controls (Fig. 4e). In addition, the level of LPS-binding protein (LBP), a surrogate marker for antigen load derived from gut bacteria^21^, and the level of interleukin-1 beta (IL-1β), a pro-inflammatory cytokine whose production is stimulated by LPS^22^, were both significantly elevated in the T1D group compared with that in the NC group (Fig. 4f, g).

Spearman’s correlation analysis between fecal metabolites and clinical parameters revealed that the concentrations of T1D-enriched metabolites, such as pterine, N1-acetylspermine, and L-pyroglutamic acid, were significantly positively correlated with serum levels of HbA1c, FBG, and TG (Fig. 4h). Additionally, T1D-reduced metabolites, such as butyric acid, acetic acid, and DL-benzylsuccinic acid, were positively correlated with butyrate-producing species, including *Faecalibacterium prausnitzii, Eubacterium rectale*, and *Roseburia faecis*, but negatively correlated with opportunistic pathogen; however, the opposite pattern was observed in case of T1D-enriched metabolites, such as pterine and L-pyroglutamic acid.

Furthermore, we constructed random forest regression models using a combination of key bacterial species and fecal metabolites to screen for T1D-associated biomarkers. The combination analysis of the key 35 bacterial species and 28 fecal metabolites revealed that a group of nine bacterial species and nine fecal metabolites provided the best discriminatory power (Fig. 4i). Notably, compared to the use of microbiome biomarkers alone, the combined markers of bacterial species and fecal metabolites provided a large improvement in the discrimination of T1D both in the discovery set (the AUC value increased from 0.815 to 0.976) and validation set (the AUC value increased from 0.649 to 0.809) (Fig. 4j). Therefore, the combination analysis could optimize the accuracy of the discrimination of T1D, and the combined biomarkers may provide novel insights into the formulation of microbiome-based strategies for the prevention and intervention of T1D.

### Disturbed glucose homeostasis induced by the transplantation of T1D-associated gut microbiota

**Fig. 5:**
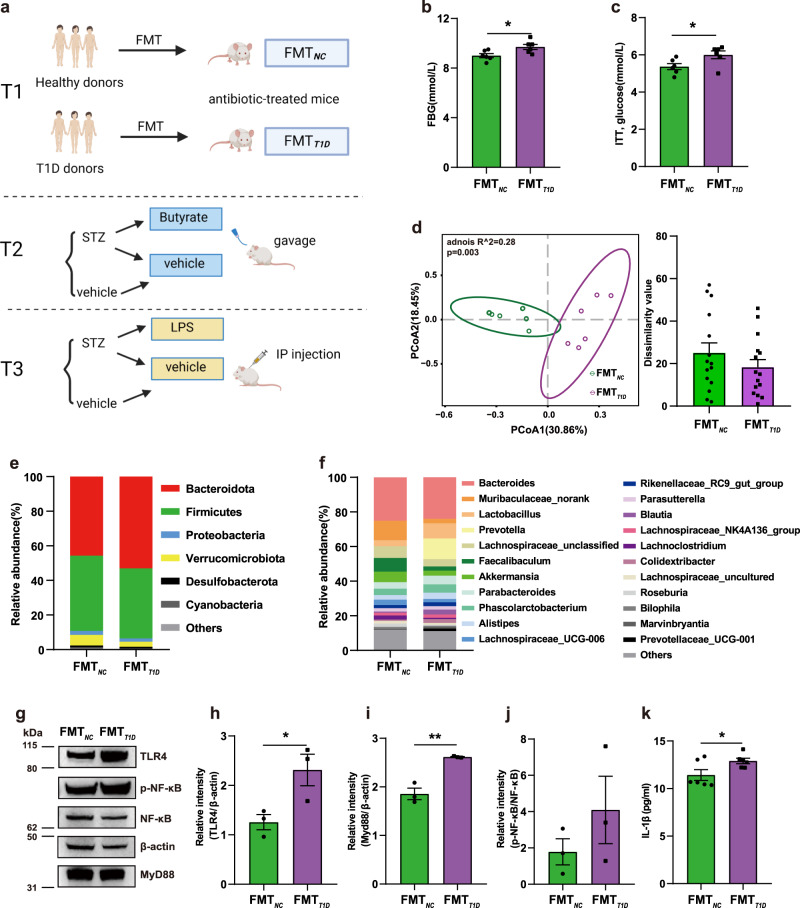
Disturbed glucose homeostasis induced by the transplantation of T1D-associated gut microbiota. **a** Schematic diagram of the study design. **b** Fasting blood glucose level (*p* = 0.025). **c** Glucose levels at 30 min after insulin injection in the ITT (*p* = 0.040). **d** PCoA plot based on the Bray-Curtis distances (left) and inner-group distance by ANOSIM analysis (right). **e**, **f** Microbial composition at the phylum (**e**) and genus (**f**) level. **g** Western blotting results depicting the TLR4, Myd88, and p-NF-κB protein expression levels in the liver. **h**–**j** Quantification of band intensities and normalization to β-actin (TLR4, *p* = 0.041; Myd88, *p* = 0.003). **k** The serum concentration of IL-1β (*p* = 0.043). FMT_*NC*_, mice recipients FMT with NC gut microbiota; FMT_*T1D*_, mice recipients FMT with T1D gut microbiota; ITT, insulin tolerance test. For **b**–**f**, **k** sample size is FMT_*NC*_: *n* = 6, FMT_*T1D*_: *n* = 6 as biologically independent samples. For **g**–**j** sample size is FMT_*NC*_: *n* = 3, FMT_*T1D*_: *n* = 3. Data represent the mean ± standard error of the mean (SEM). Unpaired two-sided *t* test. *p < 0.05, **p < 0.01. Source data are provided as a Source Data file.

To evaluate the causality between the gut microbiota and worsening of glycemic control in T1D, gut bacteria were transplanted from children with T1D and healthy children into antibiotic-treated mice (Fig. 5a and Supplementary Fig. 13). Without an external stimulus, T1D-associated mouse recipients (FMT_*T1D*_) showed higher levels of fasting glucose (Fig. 5b) compared with the controls (FMT_*NC*_). The insulin tolerance test also revealed a significantly increased glucose level after an insulin challenge of 30 min in the FMT_*T1D*_ group compared with that in the control, which indicated decreased insulin sensitivity (Fig. 5c). Interestingly, supplementation with butyrate eliminated the difference in FBG levels induced by different gut microbiota and significantly improved insulin resistance (IR) (Supplementary Fig. 14). No differences were detected between the groups with regard to the results of the oral glucose tolerance test (OGTT), insulin and C-peptide levels, and histological structure of pancreatic tissues (Supplementary Fig. 15).

We further investigated whether the differences in the gut microbiota could be transferred through FMT. The structure of the gut microbiota showed a distinct deviation between the two groups in Bray-Curtis distance-based PCoA (Fig. 5d). Alpha diversity was not significantly different between the two groups (Supplementary Fig. 16a, b). FMT_*T1D*_ mice exhibited a decreased Firmicutes/Bacteroidota ratio (Fig. 5e) and decreased relative abundance of butyrate-producing *Faecalibaculum* (Fig. 5f) compared with the controls. Moreover, the abundance of genera consisting of some beneficial bacteria, such as *Akkermansia* and *Muribaculaceae norank*, was decreased in the FMT_*T1D*_ group compared with that in the FMT_*NC*_ group.

Then, the hepatic expression of toll-like receptor 4 (TLR4), MyD88, and phosphorylated nuclear factor-kappaB (p-NF-κB) p65 was detected to evaluate the systemic inflammatory response. The hepatic relative expression of TLR4 and MyD88 was significantly increased in the FMT_*T1D*_ group compared with that in the FMT_*NC*_ group, while the ratio of p-NF-κB/NF-κB was also elevated; however, the difference was not statistically significant (Fig. 5g–j). The serum level of the pro-inflammatory cytokine IL-1β was also significantly increased in the FMT_*T1D*_ group relative to that in the controls (Fig. 5k). Therefore, these data suggested that the T1D-associated gut microbiota could induce inflammation and early symptom of diabetes.

### Roles of butyrate and LPS in streptozotocin (STZ)-induced T1D mice

**Fig. 6:**
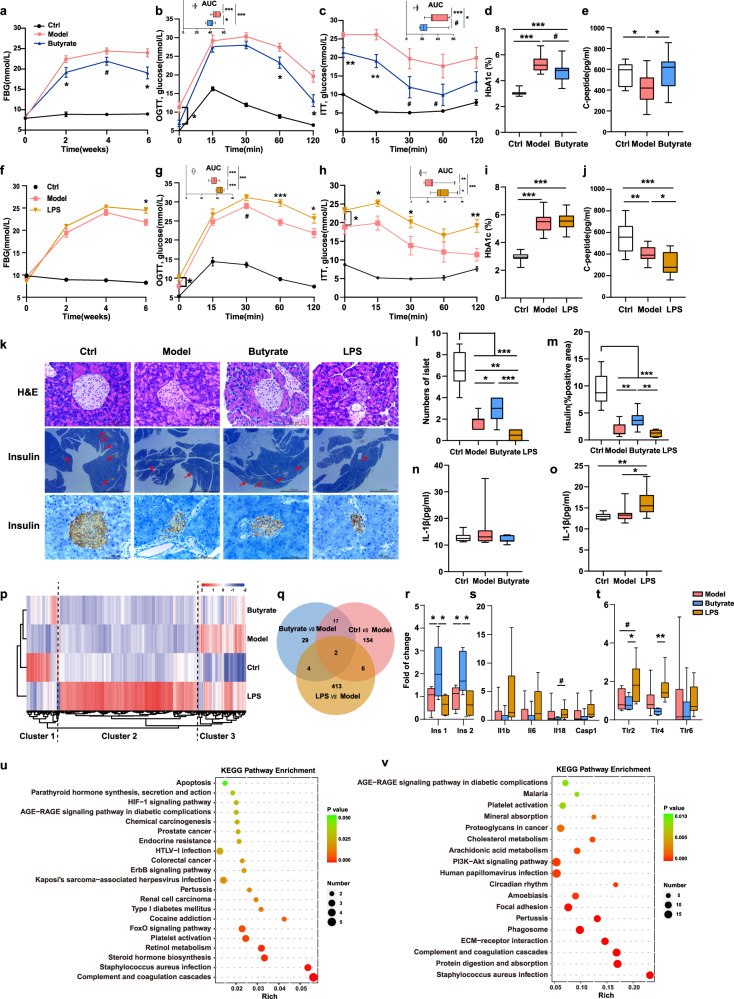
Roles of butyrate and LPS in STZ-induced T1D mice. **a** Fasting blood glucose levels (Model vs Butyrate: 2th week, *p* = 0.036; 4th week, *p* = 0.057; 6th week, *p* = 0.011), **b** Glucose tolerance test (Model vs Butyrate: 0 min, *p* = 0.022; 60 min, *p* = 0.046; 120 min, *p* = 0.012), **c** Insulin tolerance test (Model vs Butyrate: 0 min, *p* = 0.006; 15 min, *p* = 0.009; 30 min, *p* = 0.077, 60 min, *p* = 0.097), the levels of **d** HbA1c (Ctrl vs Model, *p* < 0.001; Ctrl vs Butyrate, *p* < 0.001, Model vs Butyrate, *p* = 0.053) and **e** C-peptide (Ctrl vs Model, *p* = 0.041; Model vs Butyrate, *p* = 0.026) in the experiments involving butyrate gavage. The sample size is Ctrl: n = 12, Model: *n* = 11, Butyrate: *n* = 11 as biologically independent samples. **f** Fasting blood glucose levels (Model vs LPS: 6th week, *p* = 0.011), **g** Glucose tolerance test (Model vs LPS: 0 min, *p* = 0.029; 30 min, *p* = 0.053; 60 min, *p* < 0.001; 120 min, *p* = 0.035), **h** Insulin tolerance test (Model vs LPS: 0 min, *p* = 0.035; 15 min, *p* = 0.015; 30 min, *p* = 0.044; 120 min, *p* = 0.004), the level of **i** HbA1c (Ctrl vs Model, *p* < 0.001; Ctrl vs LPS, *p* < 0.001) and **j** C-peptide (Ctrl vs Model, *p* = 0.006; Ctrl vs LPS, *p* < 0.001; Model vs LPS, *p* = 0.033) in the experiments involving LPS injection. The sample size is Ctrl: *n* = 10, Model: *n* = 12, LPS: *n* = 12 as biologically independent samples. **k** Hematoxylin-eosin (H&E) staining and immunohistochemical staining of the pancreas (40-fold and 400-fold magnification, respectively). **l** Quantification of the number of islets using the Image J software (Ctrl vs all other groups, *p* < 0.001; Model vs Butyrate, *p* = 0.030; Model vs LPS, *p* = 0.008; Butyrate vs LPS, *p* < 0.001). **m** Quantification of insulin-positive staining using Image J software (Ctrl vs all other groups, *p* < 0.001; Model vs Butyrate, *p* = 0.008; Butyrate vs LPS, *p* = 0.001). Images are representative of at least two sections per mouse, *n* = 3 mice per group. **n**, **o** The serum concentration of IL-1β in the experiments involving butyrate gavage or LPS injection (Ctrl vs LPS, *p* = 0.005; Model vs LPS, *p* = 0.021), respectively. **p** Heatmaps showing global RNA-seq expression patterns (*n* = 6 mice per group). **q** Venn diagram displaying the overlap between quantified mRNAs. **r**–**t** Pancreatic gene expression levels of **r** insulin secretion genes (*Ins1*: Model vs Butyrate, *p* = 0.046; Model vs LPS, *p *= 0.052; Butyrate vs LPS, *p* = 0.017) (*Ins2*: Model vs Butyrate, *p* = 0.022; Butyrate vs LPS, *p* = 0.010) **s** the NOD-like receptor signaling pathway (*Il 18*: Model vs Butyrate, *p* < 0.001; Butyrate vs LPS, *p* = 0.075) and **t** TLR pathway (*Tlr2*: Model vs LPS, *p* = 0.062, Butyrate vs LPS, *p* = 0.043) (*Tlr4*: Model vs Butyrate, *p* < 0.001; Model vs LPS, *p* = 0.093; Butyrate vs LPS, *p* = 0.006). **u** KEGG pathway enrichment analysis between the Model and Butyrate-treated group. **v** KEGG pathway enrichment analysis between the Model and LPS-treated group. Box and whisker plots show median ± quartiles (box), min/max (whiskers). Unpaired two-sided *t* test. *p < 0.05, **p < 0.01, ***p < 0.001, ^#^0.05 < *p* < 0.1. Source data are provided as a Source Data file.

Finally, the roles of the specific bacterial metabolites, butyrate, and LPS, were explored in STZ-induced T1D mice (Fig. 5a and Supplementary Fig. 17). Oral administration of butyrate exerted anti-diabetic effects in the T1D mouse model, as evidenced by the significantly decreased FBG, glucose levels in the OGTT and ITT, and HbA1c values compared with those in the untreated T1D mice (Fig. 6a-d). Notably, the serum C-peptide level, one of the key diagnostic criteria for T1D, increased significantly in the butyrate-treated group compared with that in the model group (Fig. 6e). Increased serum insulin levels were also observed in the butyrate-treated mice compared with those in the model group, although no statistical difference was noted (Supplementary Fig. 18a). In contrast, the LPS group showed increased FBG and glucose levels in the OGTT and ITT and decreased serum C-peptide levels compared with the model group (Fig. 6f-j and Supplementary Fig. 18c).

We then evaluated islet function. Hematoxylin-eosin staining and immunohistochemistry analysis of pancreatic sections showed that butyrate treatment alleviated the STZ-induced islet lesions with increased numbers of islets and total insulin-positive islets compared with that in the T1D mice. The LPS-treated group displayed a lower number of islets and more damaged islet structure than the model group (Fig. 6k–m). Moreover, in the butyrate-treated group, the pro-inflammatory cytokine IL-1β was reduced, although the difference was not significant, whereas LPS significantly increased the secretion of IL-1β (Fig. 6n, o), which may cause inflammatory damage to both islet structure and function. Altogether, these findings indicate that butyrate attenuates islet lesions and preserves functional beta cells, whereas LPS further aggravates islet injury and dysfunction in STZ-induced diabetic mice.

To explore the biological process and underlying mechanism, the transcriptional response to butyrate and LPS in the pancreas was monitored by RNA sequencing transcriptomic analysis. Based on the pancreatic gene expression profiles across all samples, the LPS-treated group showed a distinct profile relative to those of the other three groups. We grouped the 625 differentially expressed genes (DEGs) identified among the four groups into three clusters (Fig. 6p). Cluster 1 was mainly composed of genes associated with metabolic pathways, which were significantly enriched in the control and butyrate groups. Cluster 2 was mainly composed of pathways associated with immune and stimulus responses, which were specifically upregulated in the LPS-treated group compared with that in the remaining groups. Cluster 3 mainly consisted of pathways associated with biological processes and signaling pathways that were enriched in the model and LPS-treated groups (Fig. 6q, Supplementary Fig. 19).

**Fig. 7:**
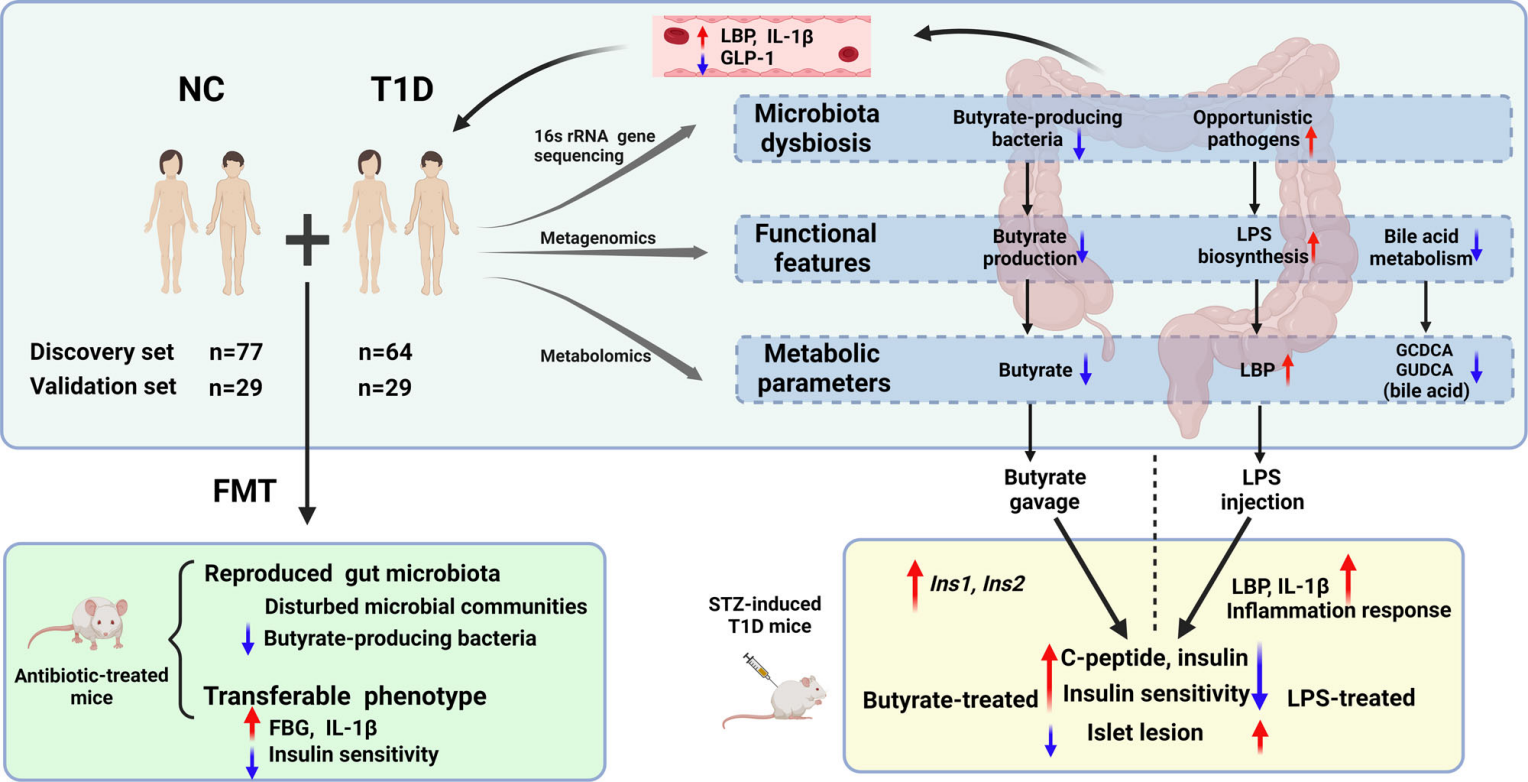
Schema summarizing key findings. In-depth multi-omics analyses revealed a deteriorated gut microbial pattern of T1D involving butyrate metabolism, LPS biosynthesis, and bile acid metabolism. The combination of 18 bacteria species and fecal metabolites as gut biomarkers excellently discriminated T1D from controls. The animal experiments further unraveled that gut microflora of T1D was a causative factor in the regulation of glucose metabolism. Butyrate and lipopolysaccharide exerted protective and destructive effects, respectively, on islet structure and function in the T1D mouse model. Created with Biorender.com.

Lastly, we identified DEGs using pairwise comparisons between the groups. In Cluster 1, we mainly focused on the key pre-proinsulin-coding genes *Insulin* (*Ins*) *1* and *Ins2*. The *Ins1* and *Ins2* expression levels were significantly higher in the butyrate-treated group, but lower in the LPS-treated group than in the model group (Fig. 6r), consistent with the results of the immunohistochemical analysis. However, representative genes in the NOD-like receptor signaling pathway (*Il1b, Il6, Il18*, and *Caspase 1*) and the TLR signaling pathway (*Tlr2, Tlr4*, and *Tlr6*) in Cluster 2 were upregulated in the LPS group but downregulated in the butyrate group compared with the case in the model group (Fig. 6s, t). KEGG pathway enrichment analysis showed that pathways related to inflammation and immune responses, including the AGE-RAGE signaling pathway in diabetic complications and phosphatidylinositol 3-kinase-Akt signaling pathway, were mainly upregulated in the LPS-treated group but downregulated in the butyrate-treated group compared with the model group. Pathways related to insulin signaling, including the forkhead box O signaling pathway, and endocrine resistance, were significantly downregulated in the butyrate-treated group compared with those in the model group (Fig. 6u, v, and Supplementary Data 10,11). A schematic overview of the study was summarized in Fig. 7.

## Discussion

Our multi-omics analyses and animal experiments deciphered the functional and metabolic profile of gut dysbiosis and explored the causal relationship and underlying mechanism between the gut microbiota and glucose dysmetabolism in T1D. The main findings are: (1) T1D-associated gut dysbiosis is characterized by increased LPS biosynthesis and decreased butyrate production and bile acid metabolism; (2) the combination of nine bacterial species and nine fecal metabolites yields excellent discriminatory power of new-onset T1D; (3) human T1D-associated gut microbiota could induce elevated fasting glucose levels and declined insulin sensitivity in antibiotic-treated mice; and 4) butyrate and LPS exert protective and destructive effects, respectively, on glucose metabolism and islet structure and function in T1D mice.

Previous genealogical^14^ and longitudinal studies^13^ of T1D cohorts have unveiled taxonomically diffuse but partly coherent functional microbial features. Comparing the microbial structures in our study with previous research, a consensus on T1D-associated microbial features at the genus or species level has not been reached. While most studies have reported a decrease in the abundance of butyrate-producing bacteria and an increase in the abundance of opportunistic pathogens from different taxa, which may be explained by “functional redundancy,” that is, species with similar functions are interchangeable in a given microbiota^23^. We profiled T1D-associated functional microbial features, including increased LPS biosynthesis, decreased butyrate production, and bile acid metabolism at the species, gene, and metabolite levels. A combination of nine fecal metabolites and nine bacterial species was identified as a T1D biomarker that yields efficiently improved discriminating performance of T1D (AUC = 0.976 and 0.809 in the discovery set and validation set, respectively), compared with that of the use of bacterial genera as biomarkers alone (AUC = 0.815 and 0.649 in the discovery and validation set, respectively). Among the nine metabolites, many are bacteria-originated or bacteria-metabolized, such as glycochenodeoxycholic acid, DL-benzylsuccinic acid, and pterine, which are significantly associated with FBG and HbA1c levels. Glycochenodeoxycholic acid, a bile acid, may play an important role in the regulation of glucose and lipid metabolism^24^. Succinate improves glucose homeostasis via intestinal gluconeogenesis^25^, whereas pterine positively correlates with inflammatory bowel disease^26^. These T1D biomarkers may reflect functional gut dysbiosis and serve as potential targets for T1D management. Our study highlights the importance of exploring disease-associated microbial features at functional and metabolic levels.

The origin of islet-specific excessive immune response in T1D remains unclear; however, the gut microbiota has been proposed as one of the culprits. LPS, an essential component of gram-negative bacteria, is a direct pathogenic factor in the induction of systemic and tissue-specific inflammatory responses^27^. Early exposure to LPS derived from different microbiota contributes to differentiated immune modulation, which affects susceptibility to T1D in humans^28^. Our integrative analysis revealed an increased relative abundance of LPS-producing bacteria and elevated expression of genes related to LPS biosynthesis in the feces of children with T1D, consistent with the elevated serum levels of inflammatory indicators, including LBP and IL-1β. Transplantation of the gut microbiota from children with T1D upregulated the expression of LPS receptor TLR4 and downstream adaptor MyD88, as well as increased serum IL-1β levels in the antibiotic-treated mice. Previous study has shown that a one-time injection of LPS into NOD mice could induce rapid inflammatory reactions in the islets^29^. Our animal experiment further revealed that the daily low-dose injection of LPS for six weeks could induce comprehensive inflammatory responses in the pancreas on the transcriptome level, aggravate islet lesion, reduce the number of insulin-positive islets and serum C-peptide level in the LPS-treated T1D mice, which may explain the disruption of glucose metabolism. Our results indicate that the LPS-mediated islet immune response may play a vital role in T1D progression; thus, blocking LPS production and entrance into the blood may be an effective therapy for T1D.

Butyrate has been widely reported to have a beneficial role in the protection of the gut barrier^30^, systematic inflammation, and glucose homeostasis^31^. The gut-derived hormone GLP-1, whose secretion is promoted by butyrate, induces glucose-dependent insulin secretion, and decreases postprandial glucagon levels^32^. We found that the levels of fecal butyrate and GLP-1 decreased in the T1D group compared with that in the control. Among all butyrate producers, *Faecalibacterium prausnitzii* contributed to 13.58% of the total abundance of genes related to butyrate metabolism, the level of which was significantly decreased in the T1D group and negatively correlated with glucose and lipid indices. *Faecalibacterium prausnitzii* regulates glucose metabolism in T1D^8,33^ and T2D^34–36^. Previous animal studies have shown that butyrate attenuates the development of necrotizing pancreatitis by reducing the serum endotoxin levels^37^ and protecting against T1D^38^. Our animal experiments further indicate that butyrate activates the expression of the *Ins1* and *Ins2* genes in the pancreas and increases insulin-positive islets and serum C-peptide levels, thus improving glucose metabolism in butyrate-treated T1D mice, relative to that in untreated mice. Our FMT experiment also showed that butyrate supplementation improves insulin sensitivity in FMT mice with T1D-associated gut microbiota. Thus, T1D is characterized by a significant decrease in the abundance of important butyrate producers, such as *Faecalibacterium prausnitzii*, and the levels of their product butyrate, which may affect islet function and histopathological integrity.

In conclusion, our multi-omics study and animal experiments extend our insights into microbial functional and metabolic dysbiosis in T1D, indicating that specific gut bacteria and metabolites may serve as novel adjuvant diagnostic, preventative, and therapeutic targets for T1D.

## Methods

The study was approved by the Institutional Review Board and Ethics Committee of Children’s Hospital of Fudan University ([2016]210, [2019]210, and [2021]181). The participants provided written informed consent; there was no financial compensation.

### Study population

This is a multi-center and cross-sectional study, centering on the Children’s Hospital of Fudan University with other top-level tertiary hospitals in nine regions in China. A total of 158 subjects in the discovery cohort and 65 in the validation cohort were initially recruited from nine regions in China from north to south area including Harbin, Changchun, Taiyuan, Jinan, Zhengzhou, Suzhou, Shanghai, Nanchang, and Fuzhou between January 2018 and July 2019. Children with T1D were firstly diagnosed according to the American Diabetes Association diagnostic criteria^39^. These children newly diagnosed with T1D were divided into two subgroups: those with DKA and those without DKA. Finally, 77 NC and 64 children with new-onset pediatric T1D were included in the discovery set, with 60.94% of T1D children experiencing DKA (*n* = 39) at T1D diagnosis. The validation set included 29 NC children and 29 children with T1D, with 58.62% of T1D children experiencing DKA (*n* = 17). Individuals were excluded if they met one of the following criteria: diagnosed with acute or chronic inflammatory diseases, infectious diseases, chronic gastrointestinal disease, other severe organic lesions, or metabolic diseases, or received antibiotics, probiotics, prebiotics, or any other medical treatment within one month.

### Sample collection

Fecal and blood samples of newly diagnosed T1D patients were collected after receiving conventional treatment for ~1 week in the hospital, to recover from metabolic decompensation, if present^40^. The control subjects were recruited from individuals who visited the outpatient clinic for health status check-ups, and sample collection was performed upon enrollment. Samples were stored at −80 °C until assayed.

### DNA extraction and 16 S rRNA gene sequencing

Fecal DNA was extracted using the Dneasy PowerSoil Kit (Qiagen, 12888-100). DNA quality and quantity were analyzed by agarose gel electrophoresis and Nanodrop 2000 spectrophotometry. DNA samples were diluted to 1 ng/μl and stored at −20 °C until further use. The total DNA was used as a template for PCR amplification for the V3-V4 regions of bacterial 16 S rRNA genes with universal primers 343 F and 798 R. The PCR-amplified library was purified using Agencourt AMPure XP beads and amplified for another round of PCR. After being purified again, the final amplicon was quantified with the Qubit dsDNA assay kit (Life Technologies, Q32852). The purified amplicon was pooled in equal amounts for sequencing. 16 s rRNA gene sequencing of the fecal DNA was performed using an Illumina MiSeq platform (MiSeq PE300, Illumina, USA) at Shanghai OE Biotech Co., Ltd.

### Bioinformatic analysis of 16 S rRNA gene sequencing data

To detect and remove the ambiguous bases, raw reads were preprocessed with Trimmomatic software (version 0.35)^41^. Next, low-quality sequences with an average quality score below 20 were trimmed with a sliding window approach. Paired-end reads were then assembled using FLASH (version 1.2.11)^42^. The assembly parameters were 10 bp minimal overlapping, 200 bp maximum overlapping, and 20% maximum mismatch rate. Reads containing ambiguous, homologous sequences or lower than 200 bp were discarded. Reads with 75% of bases above Q20 were retained and reads with chimera were removed, which were achieved with QIIME software (version 1.8.0)^43^. After the removal of primer sequences, clean reads were clustered to generate operational taxonomic units at a similarity cutoff value of 97% using the Vsearch software (version 2.4.2)^44^. Representative sequences for each OTU were obtained using the QIIME software package (version 1.8.0)^43^. Representative reads were annotated and blasted against Silva database Version 123 using an RDP classifier with a 0.70 confidence threshold^45^. Rarefaction was performed to standardize the difference in the sequencing depth.

### DNA extraction and shotgun sequencing

Total genomic DNA was extracted from fecal samples using the E.Z.N.A.® Soil DNA Kit (Omega Bio-Tek, Norcross, GA, USA). DNA concentration and purity were determined using Qubit4.0 and NanoDrop2000, respectively. Genomic DNA was fragmented to an average size of about 400 bp using Covaris M220 (Gene Company Limited, China) for paired-end library construction. The NEXTflex™ Rapid DNA-Seq Kit (Bioo Scientific, Austin, TX, USA) was used to build the paired-end library. The blunt ends of fragments were ligated with adapters containing a full complement of hybridization sites for sequencing primers. Shotgun sequencing was performed on the Illumina NovaSeq (Illumina Inc., San Diego, CA, USA) at Honsunbio Technology Co., Ltd. (Shanghai, China) with NovaSeq Reagent Kits (www.illumina.com).

### Bioinformatic analysis of shotgun sequencing data

Illumina paired-end reads were processed to remove the adaptors and low-quality reads (length <50 bp or with a quality value <20 or having N bases)^46^. Burrows-Wheeler Aligner (version 0.7.9a) aligns reads to the human genome, and any hit related to reads and their paired reads were removed^47^. Reads from the metagenomic dataset were assembled using Megahit (version 1.1.2). Finally, contigs of at least 300 bp in length were further used for gene prediction and annotation. MetaGene (http://metagene.cb.k.u-tokyo.ac.jp/) was used to predict open reading frames (ORFs) from the assembled contigs^48^. Predicted ORFs with a length being or over 100 bp were obtained and converted to amino acids using the NCBI translation table (http://www.ncbi.nlm.nih.gov/Taxonomy/taxonomyhome.html/index.cgi?chapter=tgencodes#SG1). A non-redundant gene catalog was constructed with a 90% sequence identity (90% coverage) using CD-HIT (version 4.6.1)^49^. The high-quality reads were then mapped to the non-redundant gene catalog with a 95% identity using SOAPaligner (version 2.21)^50^, and the gene abundance was calculated. Sample normalization was conducted by rarefaction and the gene abundance was normalized by the reads per kilobase per million^51^. Representative sequences of the non-redundant gene catalog were aligned to the NCBI NR database with an e-value cutoff of 1e^−5^ using Diamond (version 0.8.35)^52^. The KEGG annotation was conducted using Diamond against the KEGG database (http://www.genome.jp/kegg/) with an *e* value cutoff of 1e^−5^. The cluster of orthologous groups of proteins (COG) annotation was performed using Diamond against the eggNOG database with an e-value cutoff of 1e^−5^. CAZy annotation was conducted using hmmscan (http://hmmer.janelia.org/search/hmmscan) against the CAZy database (http://www.cazy.org/) with an e-value cutoff of 1e^−5^. Genes encoding BSHs were annotated by the UniProt database^53^. Significant differences in taxonomic and functional features between groups were performed using the Wilcoxon rank-sum test. Random-forest classifier model training was performed using the randomForest R package with 10-fold cross-validation. Cutoff values were determined by receiver operating characteristic (ROC) curve analysis. Cluster analysis was performed by hierarchical clustering using the Spearman correlation similarity measure and average linkage algorithm.

### Metabolomic profiling

Fecal metabolites were quantified by widely targeted metabolomics based on the ABSciex QTRAP®6500+ LC-MS/MS platform (Metware Biotechnology Co., Ltd., Wuhan, China). First, the fecal sample was thawed on ice. 50 mg of one sample was homogenized with 500uL of ice-cold methanol/water (70%, v/v). The samples were vortexed for 3 min, sonicated in a water bath for 10 min, and then vortexed for 1 min. After centrifugation at 12,000 rpm for 10 min at 4 °C, the supernatant was used for LC-MS/MS analysis. Then the sample extracts were analyzed based on the LC-ESI-MS/MS system (UPLC, ExionLC AD https://sciex.com.cn/; MS, QTRAP® System, https://sciex.com.cn/) and the analytical conditions were as follows: UPLC: column, Waters ACQUITY UPLC HSS T3 C18 (1.8 µm, 2.1 mm × 100 mm); solvent system, water (0.04% acetic acid): acetonitrile (0.04% acetic acid); gradient program, 95:5 V/V at 0 min, 5:95 V/V at 11.0 min, 5:95 V/V at 12.0 min, 95:5 V/V at 12.1 min, 95:5 V/V at 14.0 min; injection volume, 2 μL; flow rate, 0.4 mL/min; column temperature, 40 °C. Linear ion trap (LIT) and triple quadrupole scans were carried out based on a triple quadrupole-linear ion trap mass spectrometer (QTRAP), QTRAP® LC-MS/MS System, equipped with the ESI Turbo Ion-Spray interface, operated in a positive and negative ion mode and processed using AB SCIEX Analyst software (version 1.6.3). Operation parameters of electrospray ionization (ESI) source were set as follows: ion-spray voltage (IS) 5500 V (positive), −4500 V (negative); source temperature 500 °C; ion source gas I (GSI), gas II (GSII) and curtain gas were set at 55, 60, and 25 psi, respectively; and the collision gas (CAD) was high.

### Quantification of short-chain fatty acids

Fecal SCFA concentrations were measured by gas chromatography-mass spectrometry (GC-MS). First, fecal samples (20 mg) were weighed in a 2 ml EP tube and added 1 mL phosphoric acid (0.5% v/v) solution, then vortexed for 10 min and subjected to ultrasound for 5 min using an ultrasonic wave. After that, 0.1 mL supernatant was transferred to a new 1.5 ml tube, and 0.5 mL MTBE (containing internal standard) solution was added, then vortexed for 3 min, ultrasound for 5 min, and centrifuged for 10 min at 12000 r/min at 4 °C. After centrifugation, the 0.2 mL supernatant was collected for subsequent GC-MS analysis. The GC-MS analysis was conducted on an Agilent 7890B gas chromatography coupled to a 7000D mass spectrometer with a DB-FFAP column (30 m length × 0.25 mm i.d. × 0.25 μm film thickness, J&W Scientific, USA). The carrier gas (helium) was set at a flow rate of 1.2 mL/min, and 2 μL samples were injected in the splitless mode. The temperature program was as follows: initial oven temperature was 95 °C (held for 1 min), increased to 100 °C at a rate of 25 °C/min, then to 130 °C at a rate of 17 °C/min (held for 0.4 min), then raised to 200 °C at a rate of 25 °C/min (held for 0.5 min). The temperature was set as follows: inlet, 200 °C; transfer line, 230 °C; ion source, 230 °C; quad, 150 °C. The solvent delay was set at 3 min, the electron energy was 70 ev and all samples were analyzed using the multiple reaction monitoring modes.

### FMT experiment in an antibiotic-treated and germ-free mouse model

All animals were randomly assigned to either control or experimental groups and acclimated for 2 weeks before any experiments. C57/BL6 male mice (6 weeks old; Vital River Laboratory Animal Technology Co. Ltd., Beijing, China) were housed under specific pathogen-free conditions with food and water ad libitum at 22 °C under a 12:12 h light/dark cycle with 55–60% humidity (*n* = 6 mice per group). Mice were fed on a normal chow diet (Pu Lu Teng Biotechnology, Shanghai, China). The mice were administered filter-sterilized water supplemented with ampicillin (1 g/L), metronidazole (1 g/L), neomycin sulfate (1 g/L), and vancomycin (0.5 g/L) (Aladdin, Shanghai, China) for 2 weeks to establish the antibiotic-treated mouse model. The counts of viable bacteria in the feces of the mice were reduced by more than 90%, indicating that the antibiotic-treated animal model was successfully established. Fresh fecal samples from three children with T1D and three healthy children were randomly collected in a week and stored at −80 °C. The fecal suspension was prepared as a mixture of samples from three subjects of the same group, as previously described^54^. Lastly, 100 μL of the mixed fecal suspension was administered via oral gavage to each antibiotic-treated mouse for three consecutive days and the gavage process was repeated biweekly. Body weights and food intake were measured weekly. Fasting blood glucose (fasting for 6 h) from the tail vein blood was detected biweekly by the Accu-Chek Blood Glucose Meter (Roche). The OGTT was performed by oral gavage of 2 g/kg glucose (Sangon Biotech, Shanghai) to the mice fasted for 6 h. The insulin tolerance test was performed by intraperitoneal administration of human insulin (Novolin, 0.75 IU/kg body weight) to the mice fasted for 4 h. Mice were sacrificed at week 4. The blood samples and tissues were collected and snap-frozen.

Germ-free mice (8 weeks old, C3H/Orl male mice) were purchased from Shanghai Slack Laboratory Animal Co., Ltd (*n* = 9–10 mice per group) and were housed at the gnotobiotic facility under strict germ-free conditions. 100 μL dose of the fecal suspension was administered to each germ-free mouse by oral gavage for two consecutive days. Mice were killed in week 14.

### STZ-induced diabetic mouse

Mice were repeatedly injected intraperitoneally with low doses of STZ (Sigma-Aldrich, St. Louis, MO, USA) (50 mg/kg body weight/day) for five consecutive days to induce T1D^55^, while control mice were injected with sodium citrate buffer (*n* = 10–12 mice per group). From the first day of model establishment, mice in the butyrate-treated group were administered daily gavage with sodium butyrate (Sinopharm Chemical Reagent Co., Ltd., Shanghai, China) (500 mg/kg body weight). Mice in the LPS-treated group were intraperitoneally administered 100 µg/kg body weight LPS (L2630 from *Escherichia coli* 0111:B4, Sigma-Aldrich) daily. The control and model groups were gavaged or injected with an equal volume of normal saline or PBS in butyrate-treated and LPS-treated experiments, respectively. Mice were fasted for 12 h before the OGTT. Mice were sacrificed at week 6.

### Laboratory measurements

In the cohort study, participants’ blood HbA1c, FBG, C-peptide, WBC, NEUT, LYMPH, TC, TG, HDL-C, and LDL-C levels were measured according to uniform national quality control protocols. Serum GLP-1 (Abcam, Cambridge, UK; ab184857), FGF19 (R&D Systems, Minneapolis, MN, USA; DF1900), LBP (Panchao Biotechnology, Shanghai, China; PCDBH0284), and IL-1β (Panchao Biotechnology, PCDBH0247) concentrations were measured using enzyme-linked immunosorbent assay kits, according to the manufacturer’s instructions.

In the animal experiments, HbA1c was determined by an enzymatic method using a Hitachi 7180 Clinical Analyzer (Hitachi, Tokyo, Japan). Serum insulin (Crystal Chem Inc., 90080), C-peptide (Crystal Chem Inc., 90050), IL-1β (Abcam, ab197742), and LBP (Abcam, ab269542) concentrations were measured using the mouse ELISA kits as per the manufacturer’s instructions.

### Western blot

Total protein was extracted from the liver tissue and separated by denaturing sodium dodecyl sulfate-polyacrylamide gel electrophoresis. Proteins were then transferred onto polyvinylidene difluoride membranes (MilliporeSigma, Burlington, MA, USA). The membranes were blocked with 5% milk for 1 h and incubated overnight at 4 °C with primary antibodies against TLR4 (Proteintech Group, Rosemont, IL, USA; 19811-1-AP), MyD88 (Proteintech Group, 23230-1-AP), NF-κB p65 (Immunoway, Plano, TX, USA; YM3111), phospho-NF-κB p65 (Immunoway, YP0847), and β-actin (Immunoway, YM3028). The membranes were washed three times with phosphate-buffered saline with Tween-20 and incubated with secondary antibodies for an hour. Goat anti-rabbit IgG-HRP (Immunoway, RS0002) and goat anti-mouse IgG-HRP (Immunoway, RS0001) were used as secondary antibodies. Dilutions for primary antibodies and secondary antibodies were 1:1000 and 1:10,000, respectively. Band intensities were quantified using the Image J software (version 1.52) and normalized to β-actin levels. Original blots can be found in the accompanying Source Data file.

### Histopathology and immunohistochemistry

Pancreatic tissues were fixed with 4% paraformaldehyde and embedded in paraffin, and 3 μm sections were cut for hematoxylin-eosin staining or immunohistochemistry. Anti-insulin primary antibody (Servicebio, GB13121, 1:300) and HRP-conjugated goat anti-mouse IgG (Servicebio, GB23301, 1:200) were applied in immunohistochemistry. For quantitative analyses, randomly chosen stained pancreatic sections were used to compute the area and number of insulin-positive islets. Insulin expression was digitally quantified using the Image J software (version 1.52).

### RNA sequencing

Total RNA was isolated from mouse pancreas tissue using the Trizol reagent (Invitrogen, Waltham, MA, USA). The concentration, quality, and integrity were determined using a NanoDrop spectrophotometer (Thermo Fisher Scientific, Waltham, MA, USA). Sequencing libraries were constructed using the TruSeq RNA Sample Preparation Kit (Illumina, San Diego, CA, USA), which consisted of an mRNA purification process using poly-T oligo-attached magnetic beads, mRNA fragmentation, cDNA synthesis, end repair process, the addition of a single ‘A’ base followed by ligation of adapters, and purification and enrichment with PCR. Products were purified using the AMPure XP system (Beckman Coulter, Beverly, CA, USA) and quantified using the Agilent high sensitivity DNA assay on a Bioanalyzer 2100 system. The purified products were subjected to paired-end sequencing using the NovaSeq 6000 platform (Illumina) by Shanghai Personal Biotechnology Cp. Ltd. The quality of raw data in FASTQ format was evaluated using FastQC (version 0.11.8). Cutadapt software (version 1.15) was used to filter the sequencing data to get high-quality clean data for further analysis. HTSeq (version 0.9.1) was used to count the read counts mapped to each gene and then Fragments Per Kilo bases per Million fragments (FPKM) was used to standardize the expression. The samples were further analyzed for differential gene expression analysis and functional enrichment analysis.

### Statistics and reproducibility

No statistical method was used to predetermine the sample size. All analyses were performed blinded to the identity and clinical characteristics of the participants. For animal studies, investigators were not blinded to allocation during experiments but were blinded to outcome assessments.

The data were processed using Microsoft Excel 2019, SPSS version 21, GraphPad Prism version 8, and R version 3.5.1. Clinical characteristics were described as numbers (proportions) for categorical variables and means (standard deviations) or medians (interquartile range) for continuous variables, unless otherwise specified. A chi-square test was used to compare categorical variables. For normally distributed continuous variables, a two-tailed *t* test (unpaired) was used to compare the differences between the two groups, and the Wilcoxon rank-sum test was used if the variables were inconsistent with the normal distribution. The outliers were identified as values outside of the mean plus or minus three standard deviations (SD) and removed from further analysis. Statistical parameters, including the exact value of *n* and statistical significance (*p* value), are reported in Figure Legends. A two-sided *p* value of <0.05 was considered significant. Spearman’s correlation *p* values were corrected using the Benjamini–Hochberg false discovery rate.

### Reporting summary

Further information on research design is available in the Nature Research Reporting Summary linked to this article.

## Supplementary information

Supplementary Information

Description of Additional Supplementary Files

Supplementary Data 1-11

Reporting Summary

STORMS Checklist

## Data Availability

The source data underlying Figs. 1–6 and Supplementary Figs. 3–19 are provided as a Source Data file. The sequencing data have been deposited in the NCBI Sequence Read Archive (SRA) database (http://www.ncbi.nlm.nih.gov/sra) with the accession numbers PRJNA664632, PRJNA669199, PRJNA668202, PRJNA877820, and PRJNA868392. The metabolomics data are available in the MetaboLights database (MTBLS5898, MTBLS5919, and MTBLS5920) (http://www.ebi.ac.uk/metabolights). The datasets supporting this study are available in the Zenodo repository (10.5281/zenodo.7073918). Source data are provided in this paper.

## Source data

Source Data

## Code availability

Associated codes are available on GitHub (https://github.com/Luofhlab/Luofhlab).
