# Supplementary material for: Functional and metabolic alterations of gut microbiota in children with new-onset type 1 diabetes
Source: Nat Commun. 2022 Oct 26;13:6356. doi: 10.1038/s41467-022-33656-4 (PMC9606127; doi:10.1038/s41467-022-33656-4)
Supplement: Supplementary file 1 — Supplementary Information [file 41467_2022_33656_MOESM1_ESM.pdf]

Supplementary Information

Functional and metabolic alterations of gut microbiota in children with new-onset type 1 diabetes

Yuan et al

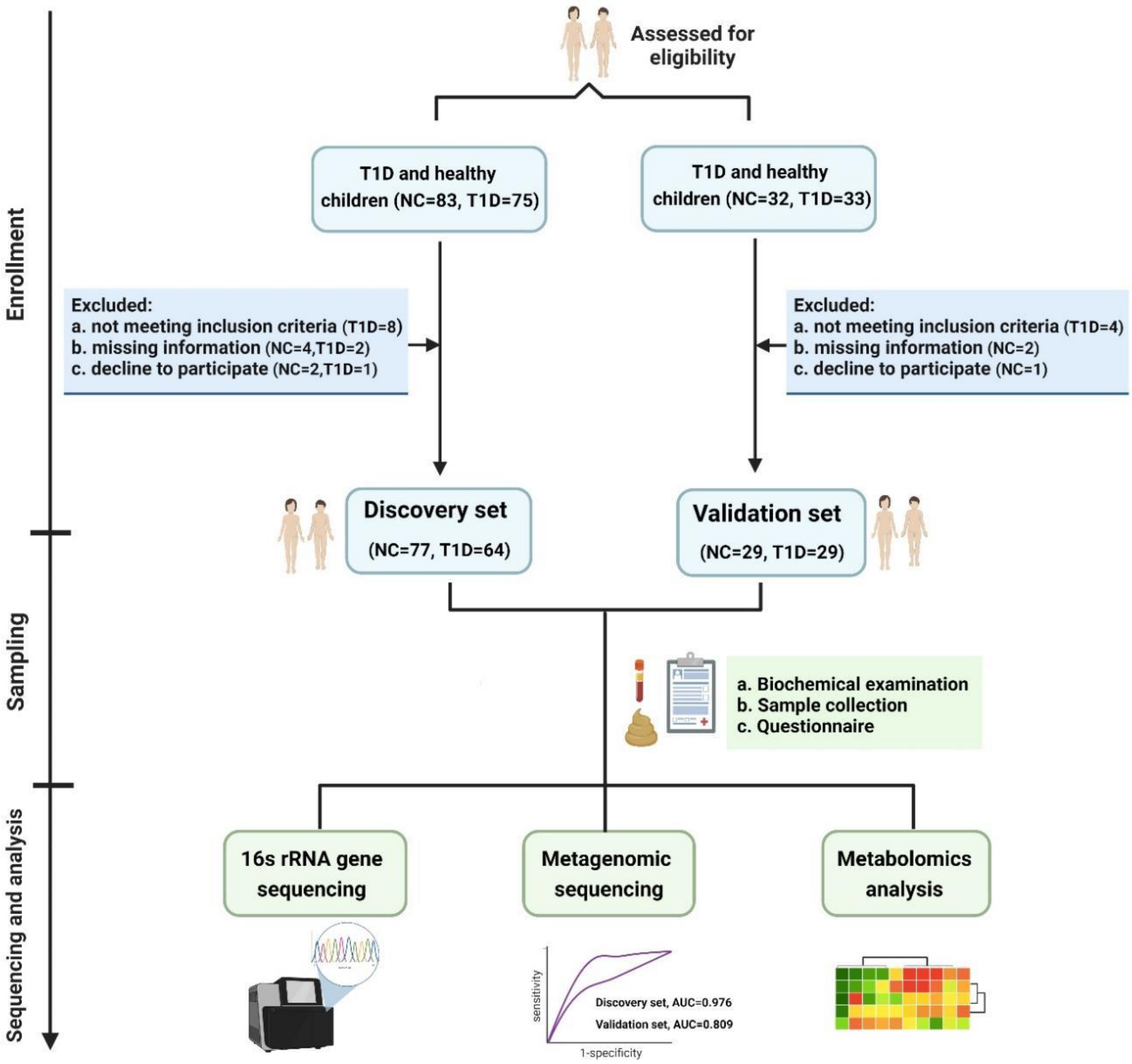

Supplementary Figure 1. Flow chart illustrating the procedures of the study. Created with BioRender.com

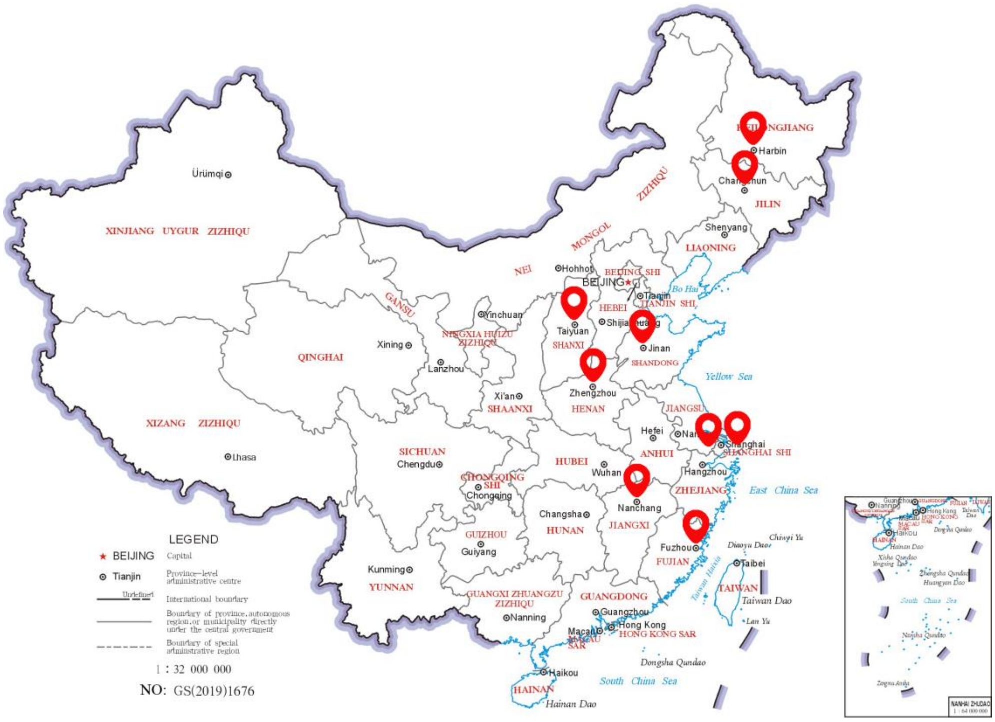

Supplementary Figure 2. Map of sampling area in this study. The red icons represent the nine sampling regions including Harbin, Changchun, Taiyuan, Jinan, Zhengzhou, Suzhou, Shanghai, Nanchang, and Fuzhou in China. The Chinese map was generated by the public standard map online service (GS (2019)1676) (http://bzdt.ch.mnr.gov.cn/).

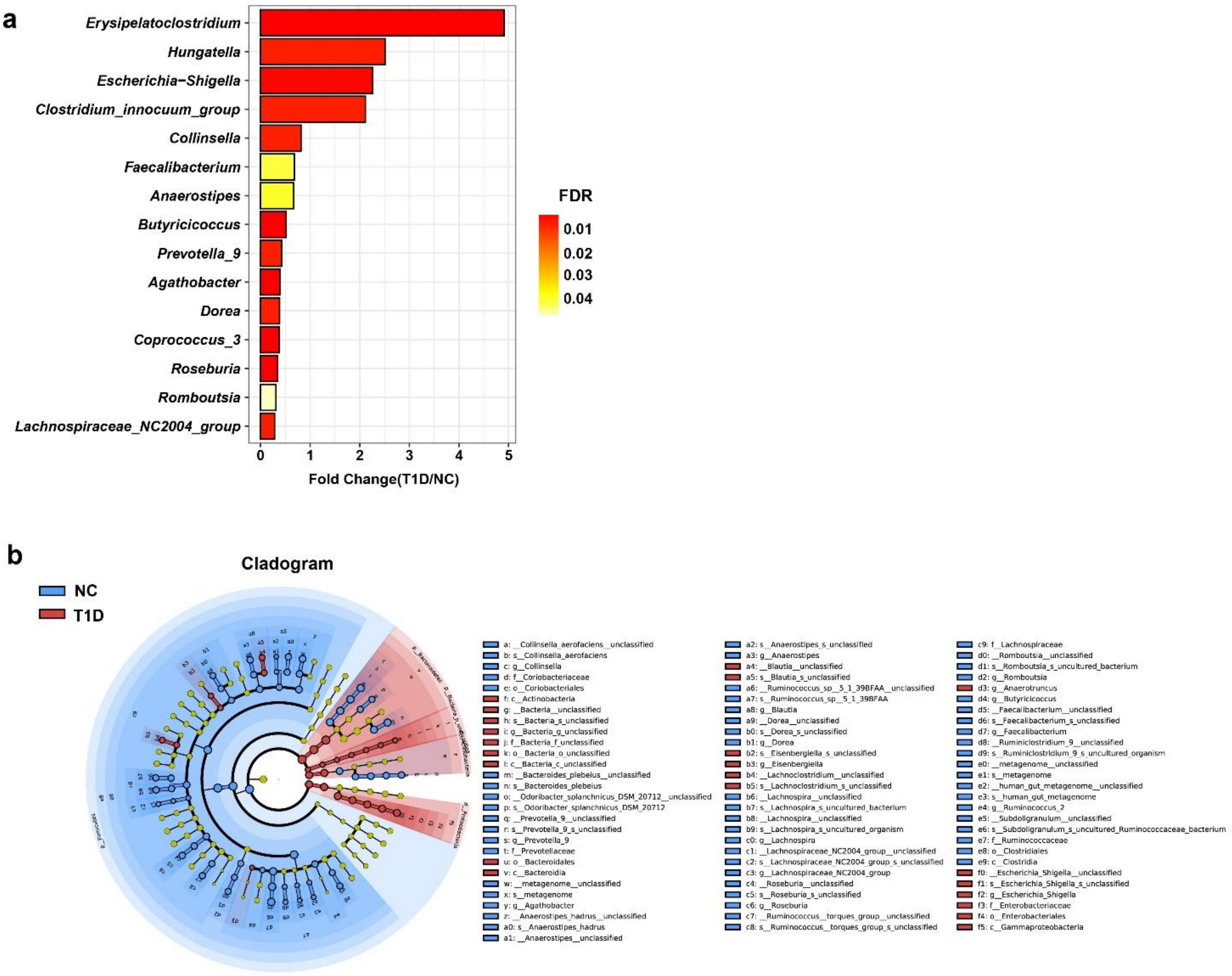

Supplementary Figure 3. Microbial communities in the discovery set. (a) Foldchange of 15 genera with the most significant differences based on the Wilcoxon rank-sum test. (b) LEfSe taxonomic cladogram of differential genera between the NC and T1D group. NC: n= 77, T1D: n = 64.

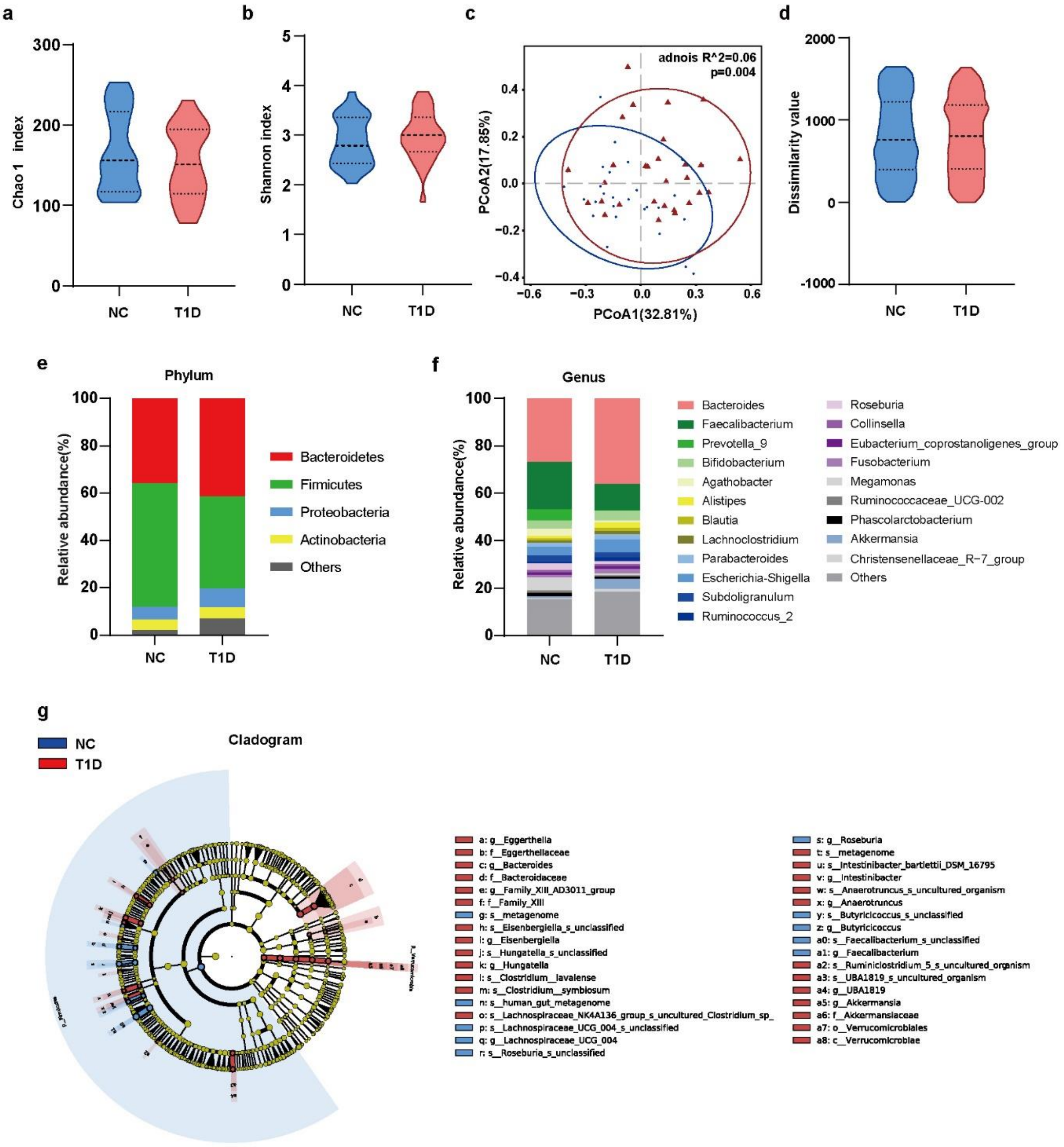

Supplementary Figure 4. The shift of gut microbiota based on 16S rRNA gene sequencing in the validation set. (a, b) The microbial community richness (Chao 1 index; a) and diversity (Shannon index; b). (c,d) PCoA analysis based on weighted Unifrac distance (c) and analysis of similarities (ANOSIM) (d). (e, f) Comparisons of relative abundance of microbial taxa at the phylum (e) and genus (f) level. (g) Cladogram generated by LEfSe analysis indicating differences in bacterial taxa between the NC and T1D group. The color of discriminative taxa represents the taxa more abundant in the corresponding group (NC in blue, T1D in red). NC: n= 29, T1D: n = 29. Violin plots show the median, quartiles, and min/max values. Two-sided Wilcoxon rank-sum test.

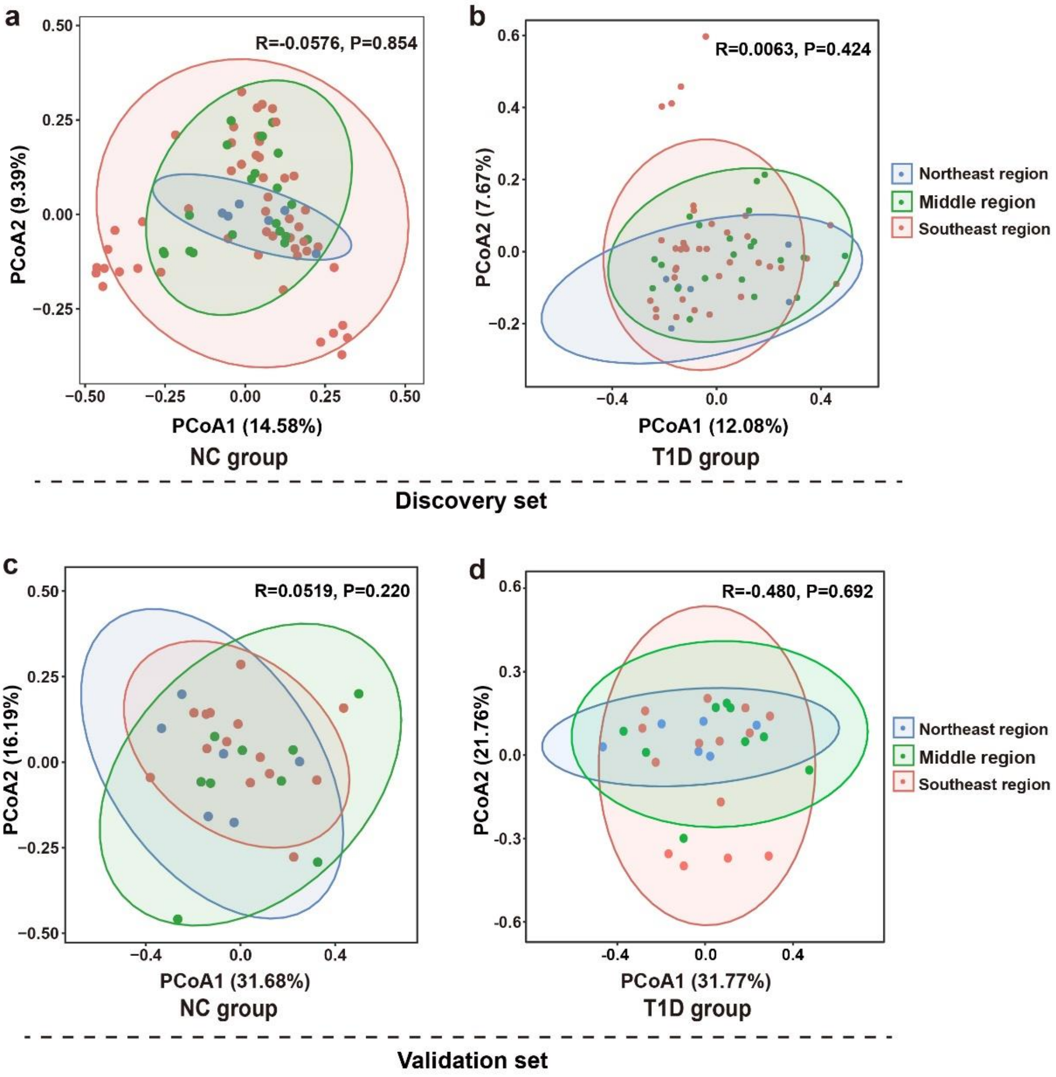

Supplementary Figure 5. PCoA of microbial communities based on the 16S rRNA gene sequencing profiles at different regions in the discovery set (a,b) and validation set (c,d), respectively. The northeast region includes Harbin and Changchun. The middle region includes Taiyuan, Jinan, and Zhengzhou. The southeast region includes Suzhou, Shanghai, Nanchang, and Fuzhou.

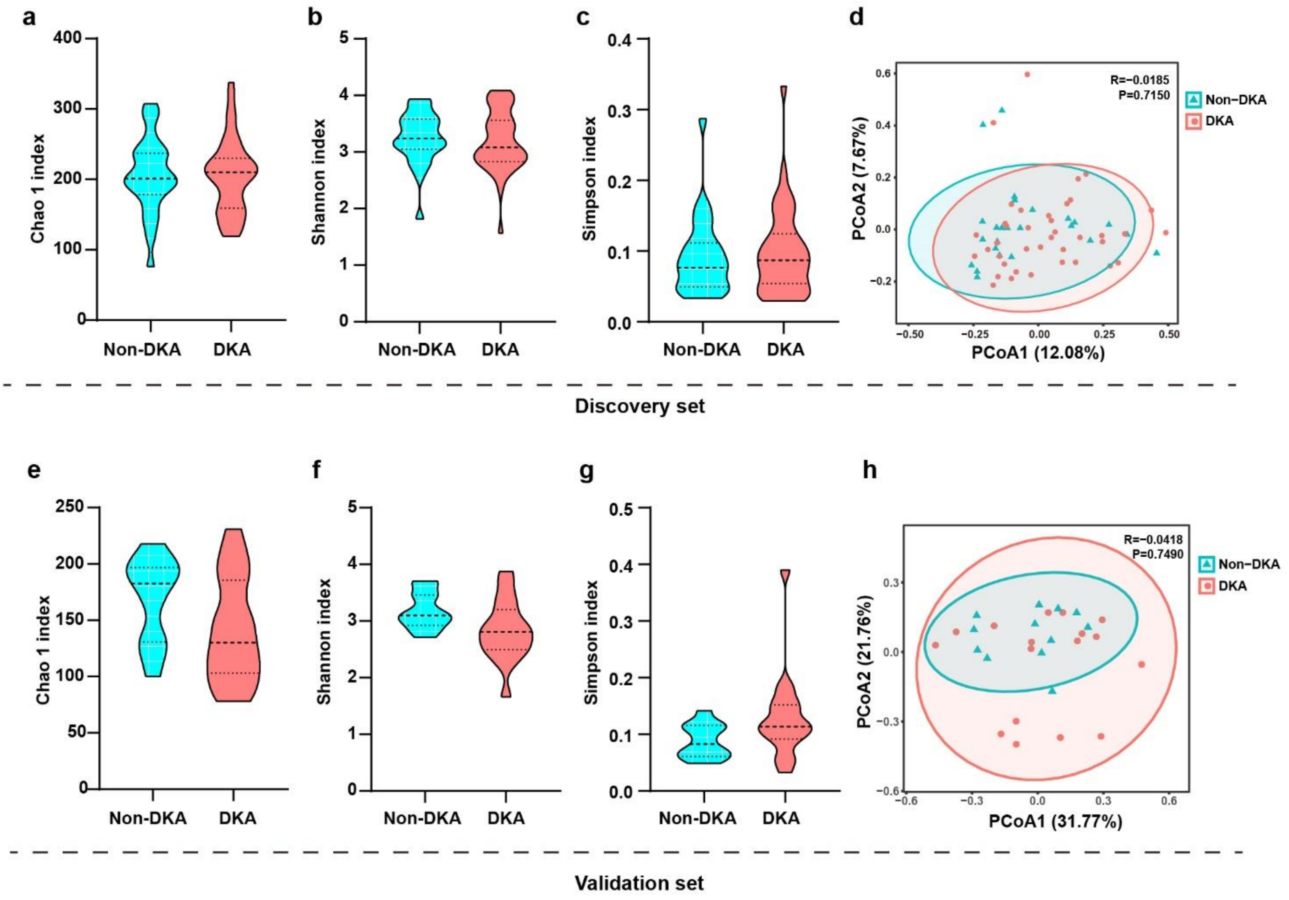

Supplementary Figure 6. Microbial communities based on the 16S rRNA gene sequencing profiles in the DKA and Non-DKA group. α diversity (Chao 1, Shannon and Simpson index) in the discovery set (a-c) and validation set (e-g), respectively. PCoA plot in the discovery set (d) and validation set (h), respectively. Violin plots show the median, quartiles, and min/max values. Two-sided Wilcoxon rank-sum test.

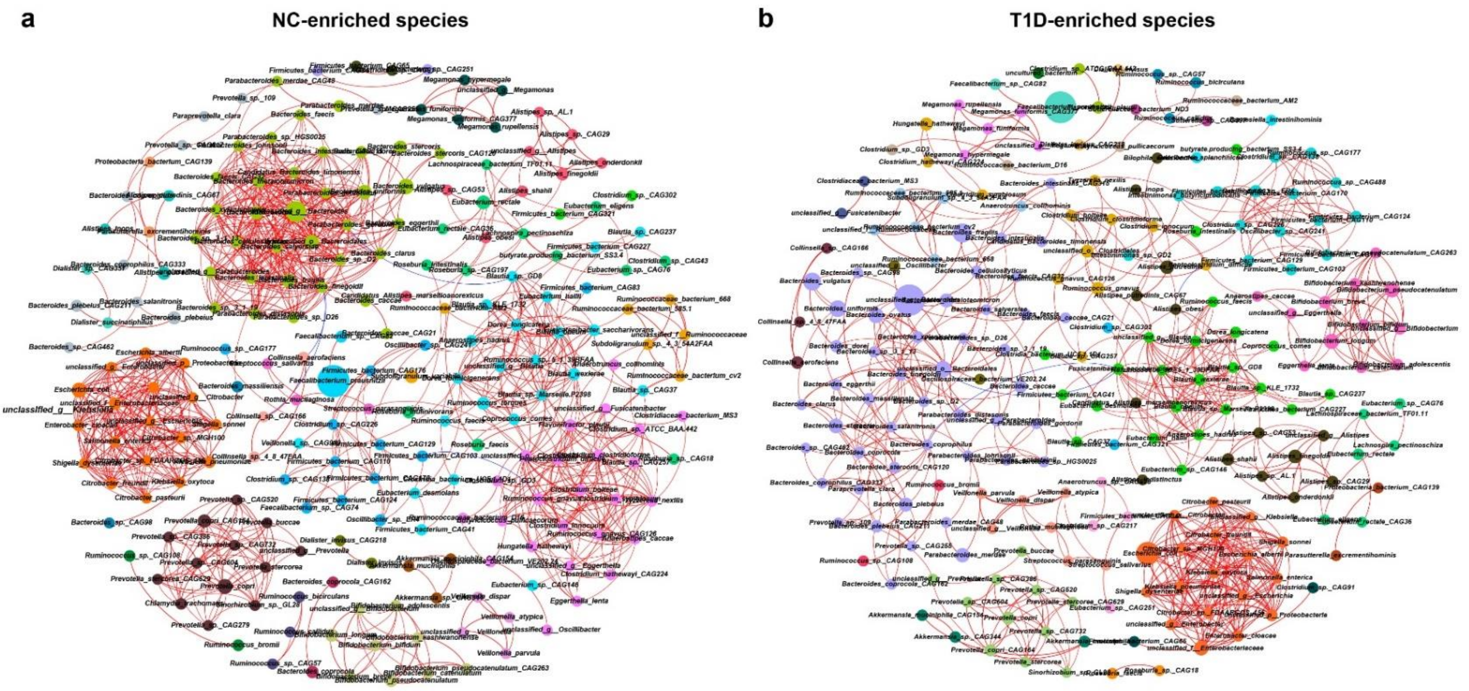

Supplementary Figure 7. The gut microbiota divergence in NC and T1D group based on the metagenomic sequencing data in the discovery set. (a, b) Co-occurrence network in the NC and T1D group based on the Spearman correlation algorithms. The node size indicates the relative abundance of each bacteria per group. Correlations were identified by the absolute value of Sparcc's coefficient > 0.60 and *p* < 0.05.

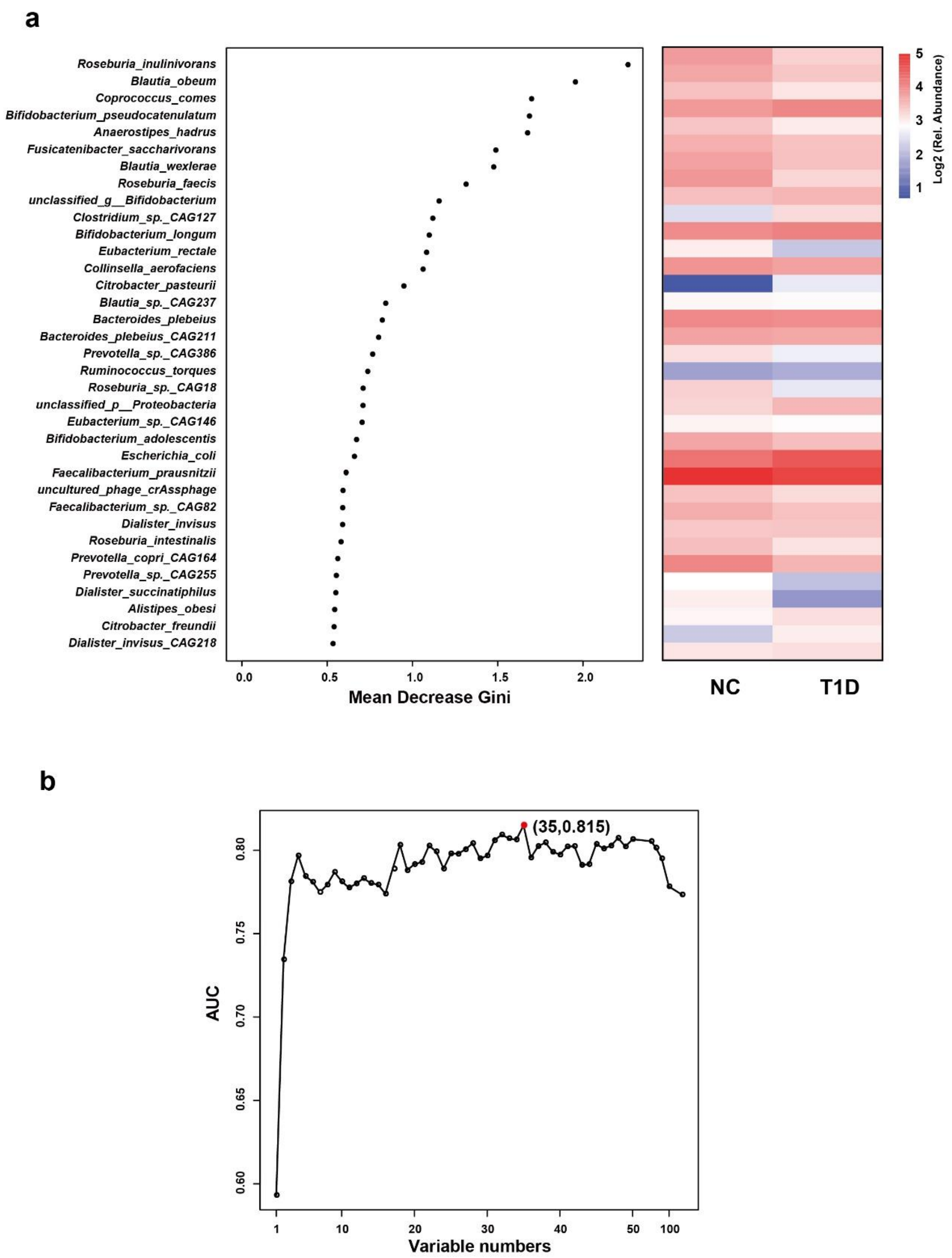

Supplementary Figure 8. Discriminatory species identified by Random Forest analysis. (a) The top 35 most important species identified by Random Forest analysis (metagenomically derived species with a relative abundance of more than 2.5% at least in one sample). (b) The area under the curve based on the cross-validation of the random forest model in the discovery set.

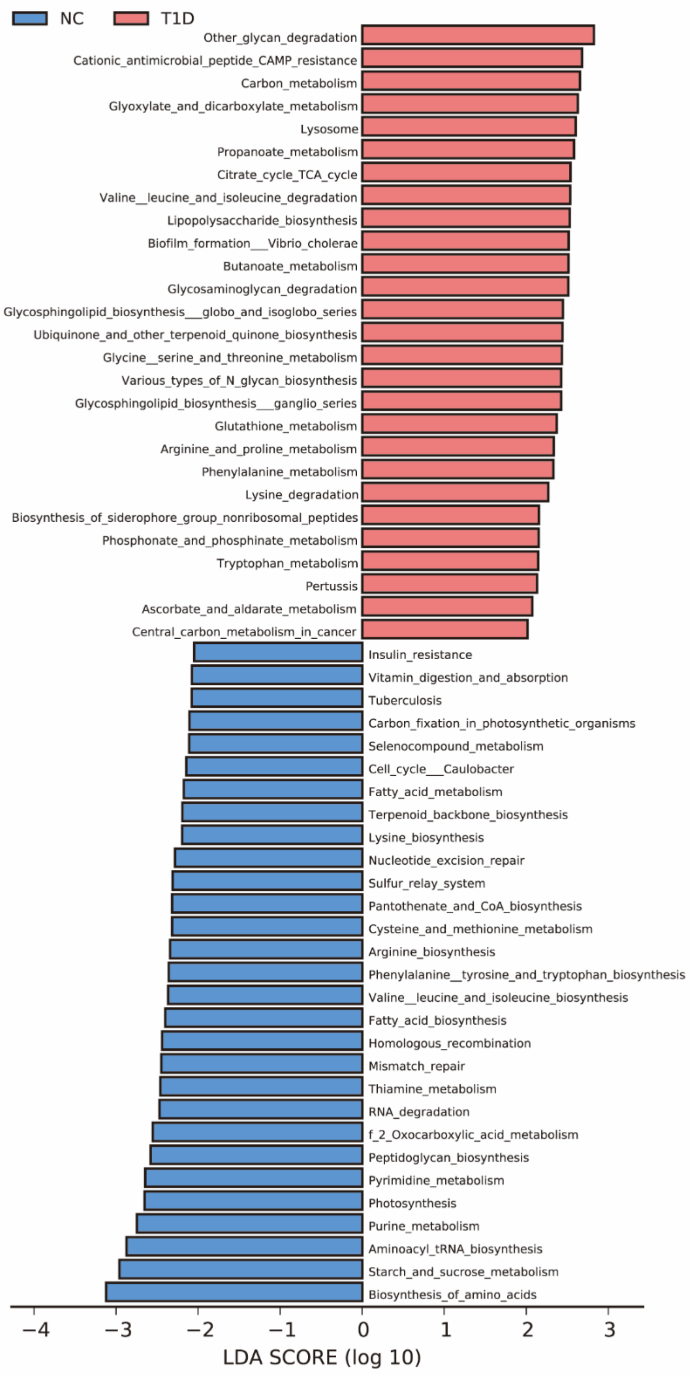

Supplementary Figure 9. The Linear discriminant analysis effect size (LEfSe) analysis for the discriminatory pathways between the NC and T1D group in the discovery set.

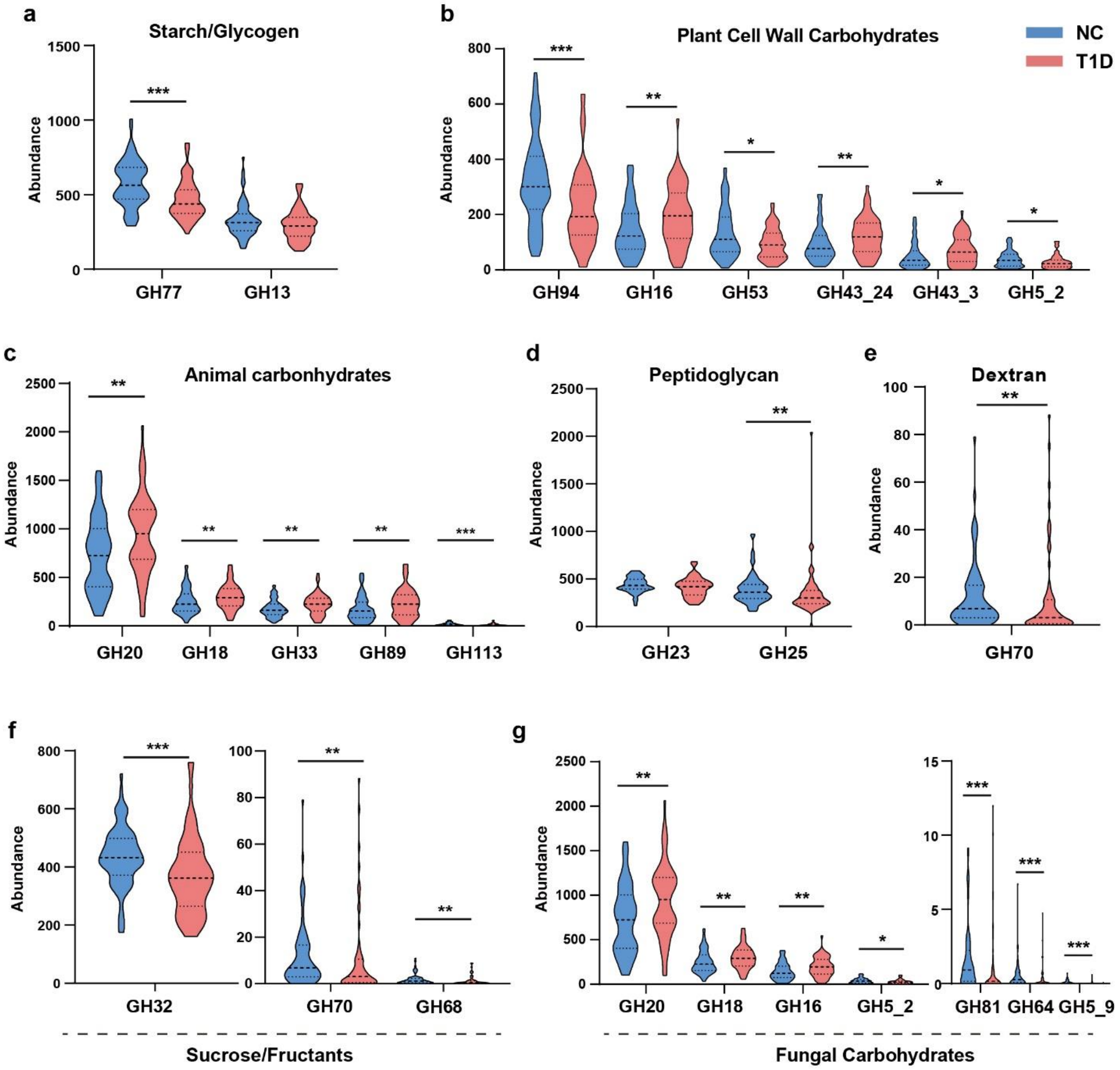

Supplementary Figure 10. The shift in the relative abundance of CAZy gene families involved in metabolizing different carbohydrate substrates in the discovery set. *GH77*, $p < 0.001$; *GH94*, $p < 0.001$; *GH16,* $p = 0.005$; *GH53,* $p = 0.026$; *GH43_24,* $p = 0.005$; *GH43_3,* $p = 0.012$; *GH5_2,* $p = 0.014$; *GH20,* $p = 0.002$; *GH18,* $p = 0.008$; *GH33,* $p = 0.004$; *GH89,* $p = 0.009$; *GH113*, $p < 0.001$; *GH25,* $p = 0.002$; *GH70,* $p = 0.005$; *GH32*, $p < 0.001$; *GH68,* $p = 0.004$; *GH81*, $p < 0.001$; *GH64*, $p < 0.001$; *GH5_9*, $p < 0.001$. Violin plots show the median, quartiles, and min/max values. Two-sided Wilcoxon rank-sum test. * $p < 0.05$, ** $p < 0.01$, *** $p < 0.001$.

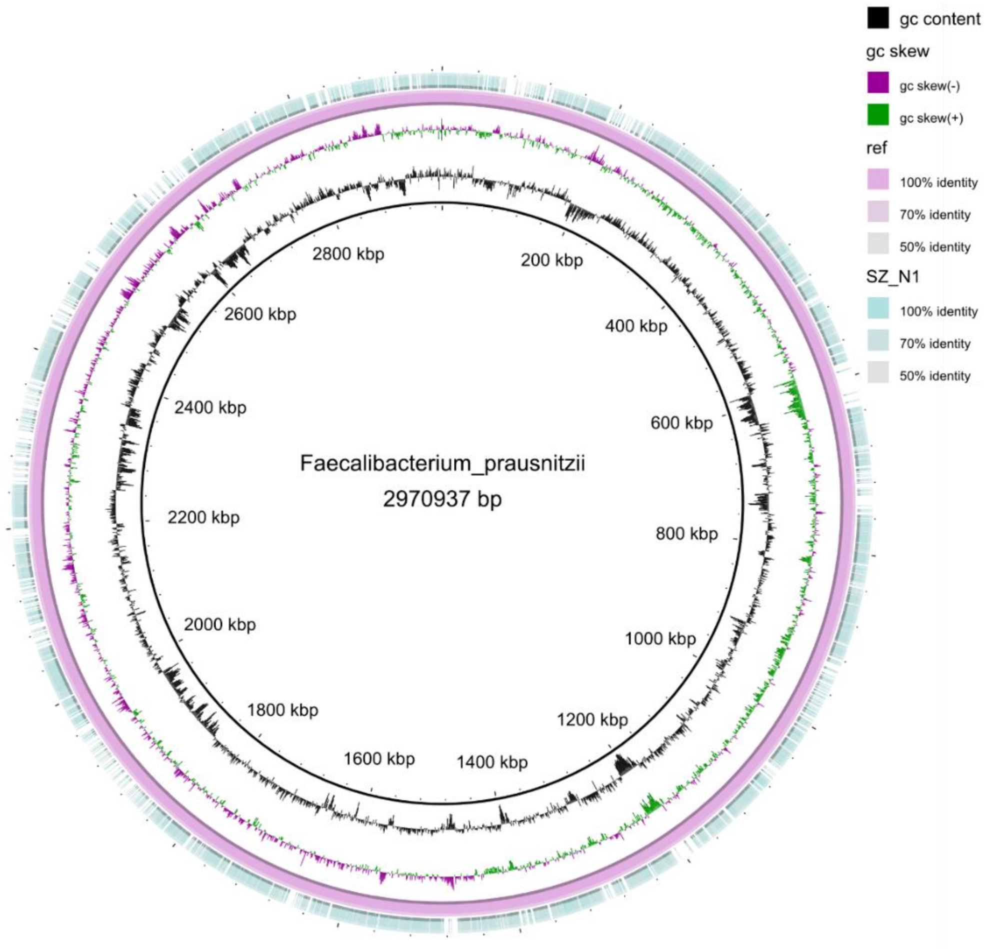

Supplementary Figure 11. High-quality draft genome of *Faecalibacterium prausnitzii*. SZ_N1 is the representative sample with the highest relative abundance for *Faecalibacterium prausnitzii*.

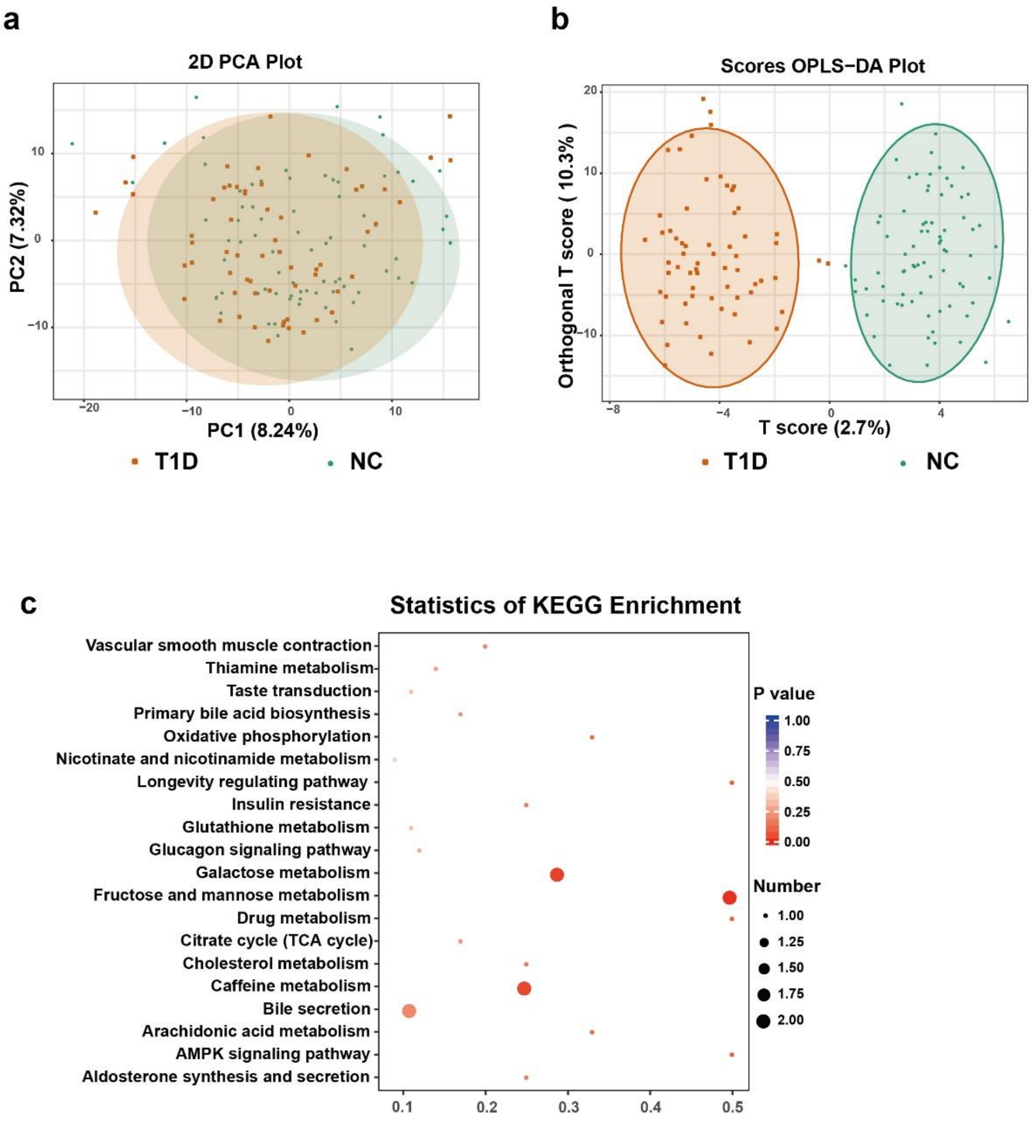

Supplementary Figure 12. Fecal metabolic profiles. (a) PCA scores on samples between the NC and T1D group. (b) The analyses of orthogonal partial least-squares discriminant analysis (OPLS-DA). (c) KEGG pathway enrichment analysis between the NC and T1D group.

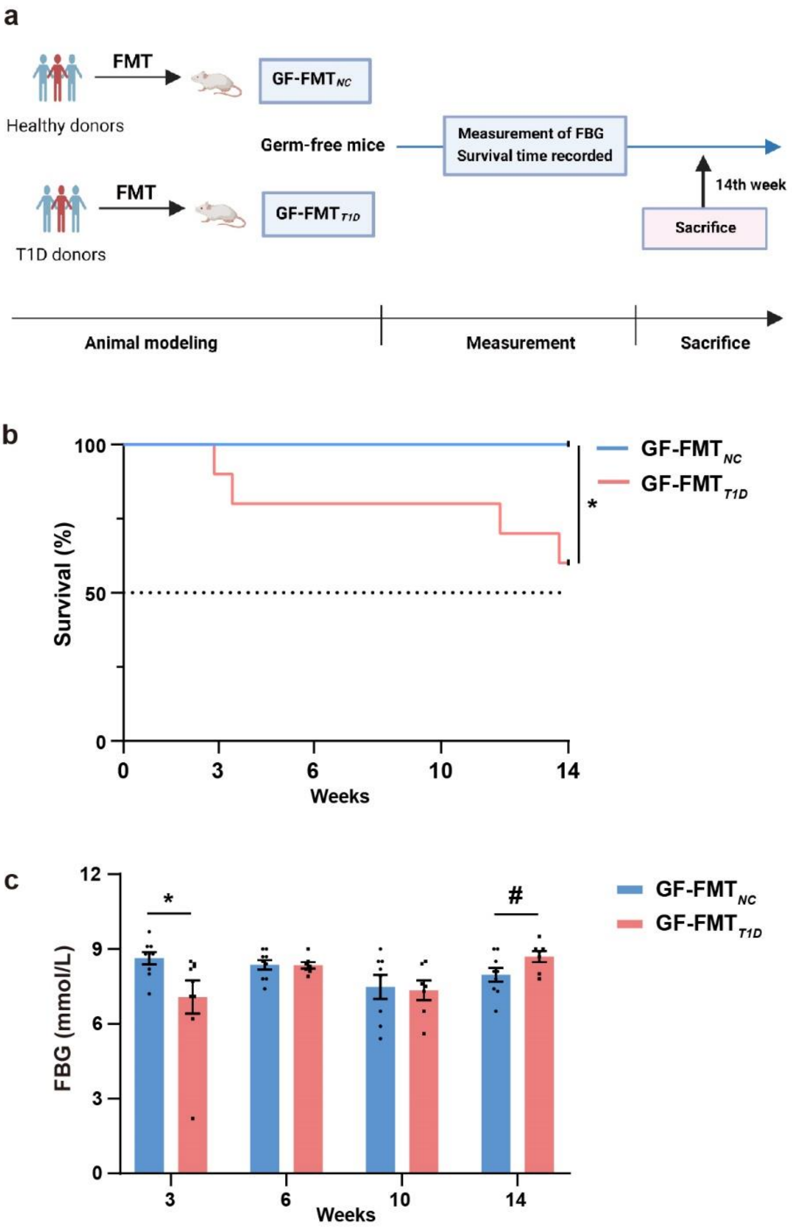

Supplementary Figure 13. FMT with NC and T1D-associated gut microbiota in germ-free mice. (a) Schematic diagram of the study design. (b) Survival analysis of GF-FMT$_{NC}$ (n=9) and GF-FMT$_{T1D}$ (n=10) after FMT (p = 0.038, Log-rank test) . (c) Fasting blood glucose level after FMT. 3th week, p = 0.042; 14th week, p = 0.091. Data represent the mean ± SEM. Unpaired two-tailed t-test. * $p < 0.05$, # $0.05 < p < 0.1$.

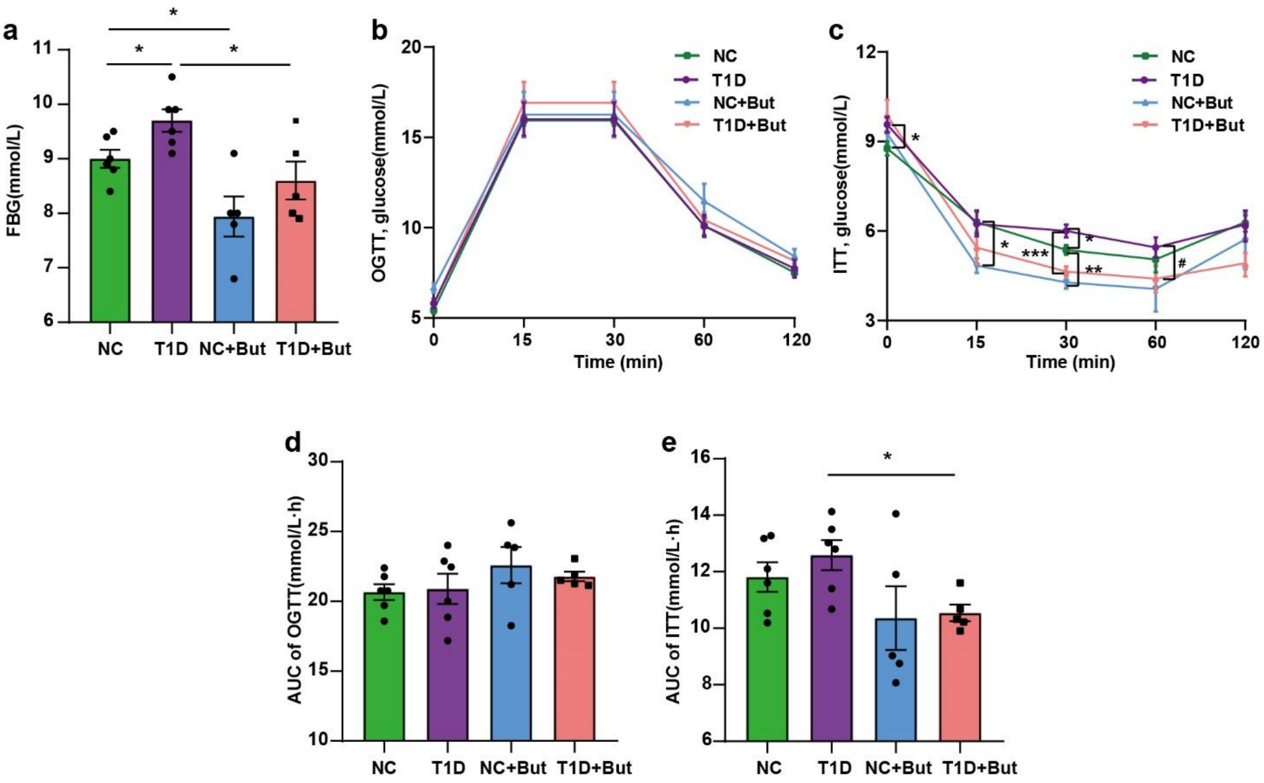

Supplementary Figure 14. FMT with NC and T1D-associated gut microbiota in antibiotic-treated mice with the supplement of butyrate. (a) Fasting blood glucose levels in four groups (NC vs T1D, $p = 0.025$; NC vs NC+But, $p = 0.020$; T1D vs T1D+But, $p = 0.019$). (b, c) Glucose tolerance test (b) and Insulin tolerance test (c) (0min, NC vs T1D, $p = 0.046$; 15min, NC vs NC+But, $p = 0.015$; 30min, NC vs T1D, $p = 0.040$; NC vs NC+But, $p = 0.003$; T1D vs T1D+But, $p < 0.001$; 60min, T1D vs T1D+But, $p = 0.094$). (d, e) The area under the curve (AUC) in the OGTT (d) and ITT (e) (T1D vs T1D+But, $p = 0.011$). NC, mice recipients FMT with NC gut microbiota (n=6); T1D, mice recipients FMT with T1D gut microbiota (n=6); NC+But, NC mice recipients gavaged by butyrate (n=5); T1D+But, T1D mice recipients gavaged by butyrate (n=5). Data represent the mean ± SEM. Unpaired two-tailed t-test. * $p < 0.05$, ** $p < 0.01$, *** $p < 0.001$, # $0.05 < p < 0.1$.

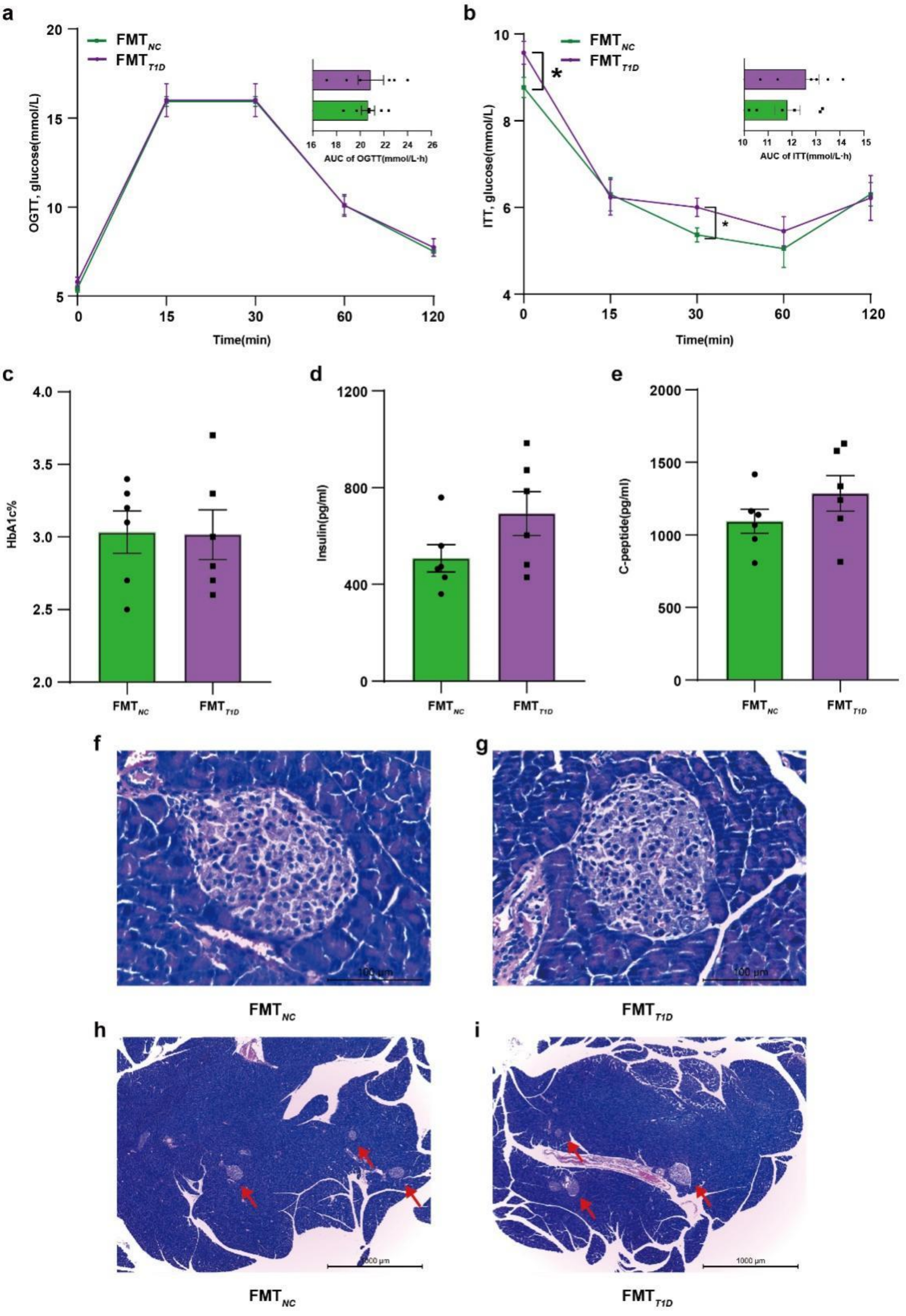

Supplementary Figure 15. FMT with NC and T1D-associated gut microbiota in antibiotic-treated mice. (a, b) Glucose tolerance test and Insulin tolerance test (0min, p = 0.046; 30min, p = 0.040) with AUC. (c-e) The level of HbA1c (c), insulin (d), and C-peptide (e) in the FMT$_{NC}$ (n=6) and FMT$_{T1D}$ (n=6) group. (f-i) HE staining of the pancreas in two groups (400-fold and 40-fold magnification, respectively). Data represent the mean ± SEM. Unpaired two-tailed t-test. * $p < 0.05$.

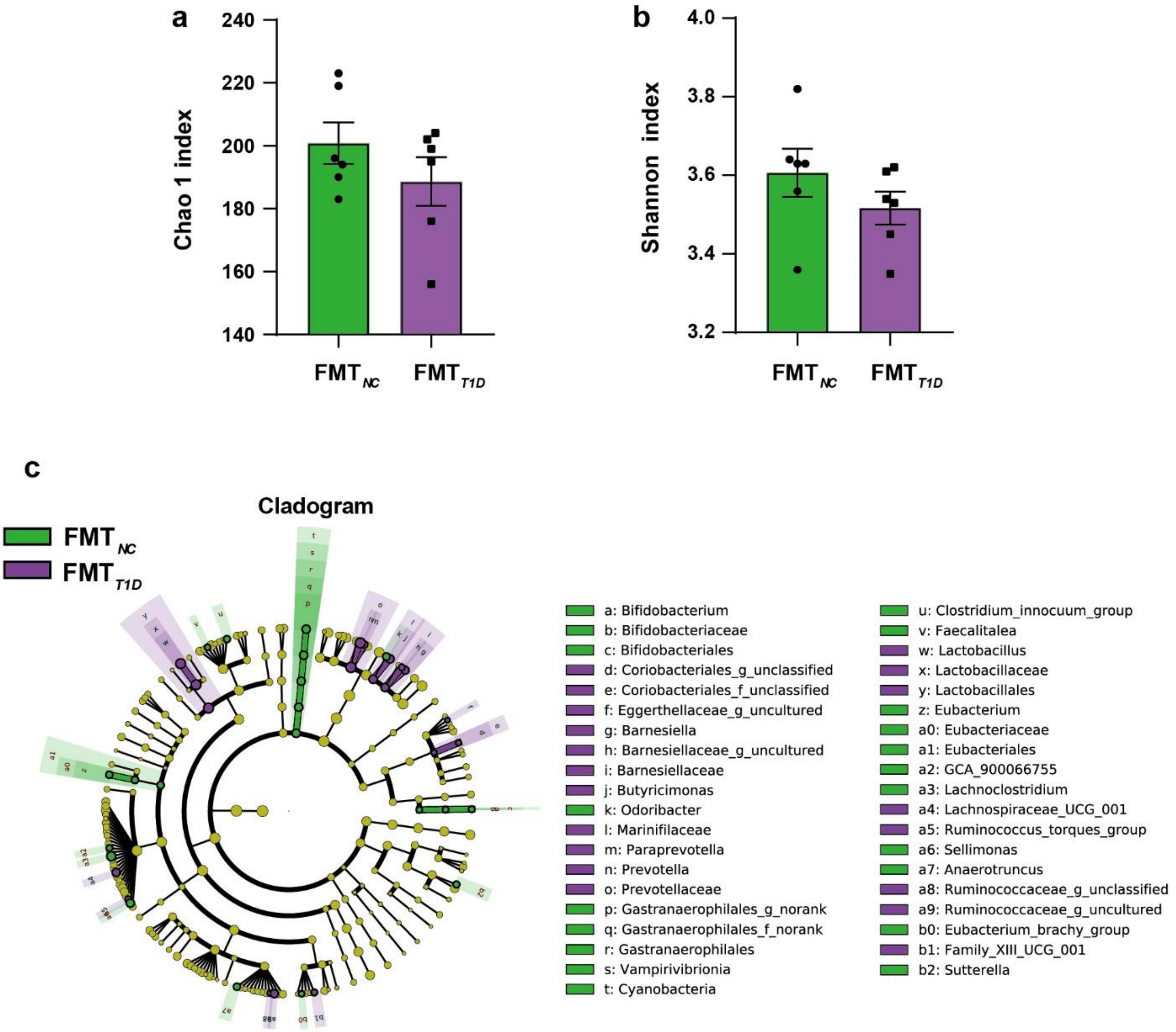

Supplementary Figure 16. The shift of gut microbiota based on 16S rRNA gene sequencing in the FMT_{NC} and FMT_{T1D} groups. (a, b) The microbial community richness (Chao 1 index; a) and diversity (Shannon index; b). (c) Cladogram generated by LEfSe showing differences in bacterial taxa between the FMT_{NC} and FMT_{T1D} groups. The color of discriminative taxa represents the taxa that are more abundant in the corresponding group (FMT_{NC} in green, FMT_{T1D} in purple). n = 6 mice per group. Data represent the mean ± SEM. Unpaired two-tailed t-test.

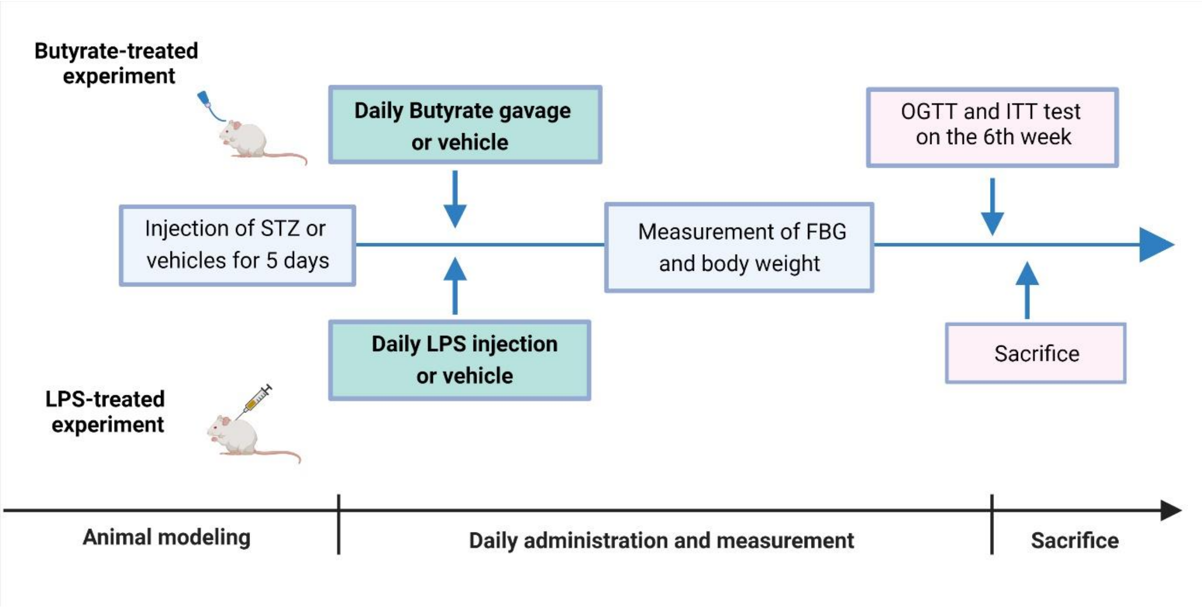

Supplementary Figure 17. The animal experimental flowchart in butyrate-treated and LPS-treated experiments.

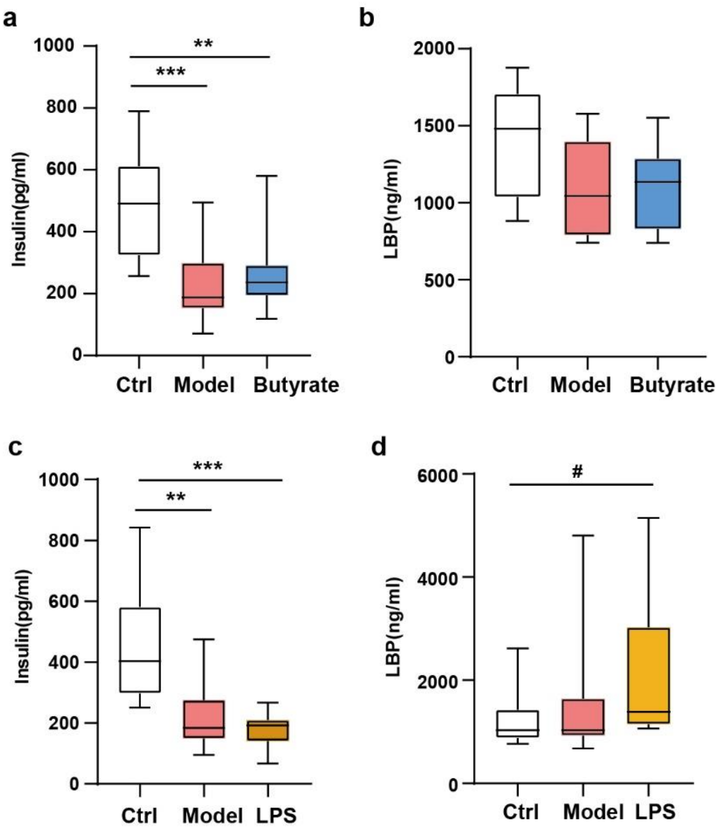

Supplementary Figure 18. Serum insulin and LBP levels in butyrate-treated and LPS-treated experiments. (a, b) Serum insulin (a) (Ctrl vs Model, $p < 0.001$; Ctrl vs Butyrate, $p = 0.002$) and LBP (b) levels in the butyrate-treated experiments. (c, d) Serum insulin (c) (Ctrl vs Model, $p = 0.001$; Ctrl vs LPS, $p < 0.001$) and LBP (d) levels (Ctrl vs LPS, $p = 0.080$) in the LPS-treated experiments. Ctrl, control group; Model, STZ-induced T1D group; Butyrate, STZ-induced T1D mice gavaged by butyrate; LPS, STZ-induced T1D mice injected with LPS. In the butyrate-treated experiment, Ctrl:n = 10, Model: n = 11, Butyrate: n=11; in the LPS-treated experiment, Ctrl: n = 10, Model: n = 12, LPS: n=11. Box and whisker plots show median ± quartiles (box), min/max (whiskers). Unpaired two-sided t-test. ** $p < 0.01$, *** $p < 0.001$, # $0.05 < p < 0.1$.

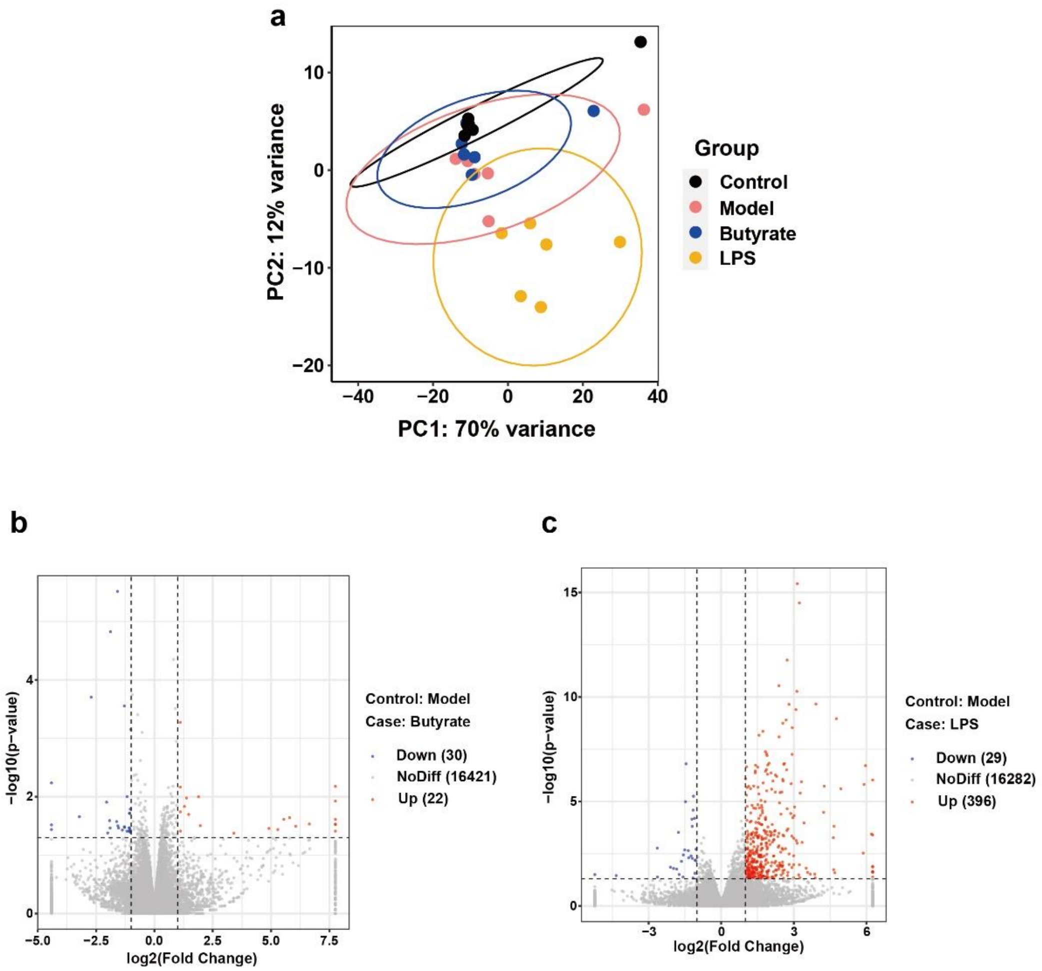

Supplementary Figure 19. RNA-sequencing analysis in butyrate-treated and LPS-treated experiments. (a) Principal component analysis (PCA) plot of RNA-seq datasets. (b, c) Volcano plot of differentially expressed genes in butyrate-treated and LPS-treated experiments, respectively.
