# Supplementary material for: Functional and metabolic alterations of gut microbiota in children with new-onset type 1 diabetes
Source: Nat Commun. 2022 Oct 26;13:6356. doi: 10.1038/s41467-022-33656-4 (PMC9606127; doi:10.1038/s41467-022-33656-4)
Supplement: Supplementary file 2 — Description of Additional Supplementary Files [file 41467_2022_33656_MOESM2_ESM.docx]

**Description of Additional Supplementary Files**

File Name: Supplementary Data 1

Description: The importance score identified by the random forest classifier model.

File Name: Supplementary Data 2

Description: Information on metagenomic sequencing data.

File Name: Supplementary Data 3

Description: Species annotated by Diamond in the NC and T1D group.

File Name: Supplementary Data 4

Description: Differences in the abundance of KEGG Orthologs (KOs) between the NC and T1D group. Two-sided Wilcoxon rank-sum test.

File Name: Supplementary Data 5

Description: Annotation of carbohydrate-related enzymes. Two-sided Wilcoxon rank-sum test.

File Name: Supplementary Data 6

Description: Genes involved in butyrate production. Two-sided Wilcoxon rank-sum test.

File Name: Supplementary Data 7

Description: Butyrate-producing gene annotation results of *Feacalibacterium prausnitzii*.

File Name: Supplementary Data 8

Description: The gene abundance of microbial bile salt hydrolases (BSHs). Two-sided Wilcoxon rank-sum test.

File Name: Supplementary Data 9

Description: Summary of widely-targeted metabolomics profiling in the NC and T1D group.

File Name: Supplementary Data 10

Description: KEGG enrichment analysis of differentially expressed genes (DEGs) in the Butyrate group compared with the Model group. *P-*value was calculated using the hypergeometric distribution.

File Name: Supplementary Data 11

Description: KEGG enrichment analysis of differentially expressed genes in the LPS group compared with the Model group. *P*-value was calculated using the hypergeometric distribution.
