# Supplementary material for: Functional and metabolic alterations of gut microbiota in children with new-onset type 1 diabetes
Source: Nat Commun. 2022 Oct 26;13:6356. doi: 10.1038/s41467-022-33656-4 (PMC9606127; doi:10.1038/s41467-022-33656-4)
Supplement: Supplementary file 4 — Reporting Summary [file 41467_2022_33656_MOESM4_ESM.pdf]

nature research

Corresponding author(s): Feihong Luo

Last updated by author(s): Sep 15, 2022

Reporting Summary

Nature Research wishes to improve the reproducibility of the work that we publish. This form provides structure for consistency and transparency in reporting. For further information on Nature Research policies, see our Editorial Policies and the Editorial Policy Checklist.

Statistics

For all statistical analyses, confirm that the following items are present in the figure legend, table legend, main text, or Methods section.

n/a	Confirmed	
☐	☒	The exact sample size (n) for each experimental group/condition, given as a discrete number and unit of measurement
☐	☒	A statement on whether measurements were taken from distinct samples or whether the same sample was measured repeatedly
☐	☒	The statistical test(s) used AND whether they are one- or two-sided<br>*Only common tests should be described solely by name; describe more complex techniques in the Methods section.*
☒	☐	A description of all covariates tested
☐	☒	A description of any assumptions or corrections, such as tests of normality and adjustment for multiple comparisons
☐	☒	A full description of the statistical parameters including central tendency (e.g. means) or other basic estimates (e.g. regression coefficient) AND variation (e.g. standard deviation) or associated estimates of uncertainty (e.g. confidence intervals)
☐	☒	For null hypothesis testing, the test statistic (e.g. F, t, r) with confidence intervals, effect sizes, degrees of freedom and P value noted<br>*Give P values as exact values whenever suitable.*
☒	☐	For Bayesian analysis, information on the choice of priors and Markov chain Monte Carlo settings
☐	☒	For hierarchical and complex designs, identification of the appropriate level for tests and full reporting of outcomes
☐	☒	Estimates of effect sizes (e.g. Cohen's d, Pearson's r), indicating how they were calculated

Our web collection on statistics for biologists contains articles on many of the points above.

Software and code

Policy information about availability of computer code

Data collection	Illumina MiSeq platform (MiSeq PE300, Illumina, USA), Illumina NovaSeq (Illumina Inc., San Diego, CA, USA), ABSciex Q-TRAP®6500+ LC-MS/MS platform (Metware Biotechnology Co., Ltd., Wuhan, China), NovaSeq 6000 platform (Illumina).
Data analysis	Microsoft Excel 2019, SPSS version 21, GraphPad Prism version 8, and R version 3.5.1, Trimmomatic software (version 0.35), FLASH (version 1.2.11), QIIME software (version 1.8.0), Vsearch software (version 2.4.2), fastp (version 0.20.0), BWA (version 0.7.9a), MEGAHIT(version 1.1.2), MetaGene (http://metagene.cb.k.u-tokyo.ac.jp/), CD-HIT(version 4.6.1), SOAPaligner (version 2.21), Diamond (version 0.8.35), Analyst 1.6.3 software (Sciex), ImageJ software (version 1.52), Cutadapt software (version 1.15), HTSeq (version 0.9.1), LEfSe tool (hhttps://huttenhower.sph.harvard.edu/galaxy).

For manuscripts utilizing custom algorithms or software that are central to the research but not yet described in published literature, software must be made available to editors and reviewers. We strongly encourage code deposition in a community repository (e.g. GitHub). See the Nature Research guidelines for submitting code & software for further information.

Data

Policy information about availability of data

All manuscripts must include a data availability statement. This statement should provide the following information, where applicable:

- Accession codes, unique identifiers, or web links for publicly available datasets
- A list of figures that have associated raw data
- A description of any restrictions on data availability

The source data underlying Fig. 1-6 and Supplementary Fig. 3-19 are provided as a Source Data file. The sequencing data have been deposited in the NCBI Sequence Read Archive (SRA) database (http://www.ncbi.nlm.nih.gov/sra) with the accession numbers PRJNA664632, PRJNA669199, PRJNA668202, PRJNA877820 and PRJNA868392. The metabolomics data are available in the MetaboLights database (MTBLS5898, MTBLS5919 and MTBLS5920) (http://www.ebi.ac.uk/metabolights).

The datasets supporting this study are available in the Zenodo repository (DOI: 10.5281/zenodo.7073918). Source data are provided with this paper. Associated codes are available on GitHub (https://github.com/Luofhlab/Luofhlab).

Field-specific reporting

Please select the one below that is the best fit for your research. If you are not sure, read the appropriate sections before making your selection.

- [x] Life sciences
- [ ] Behavioural & social sciences
- [ ] Ecological, evolutionary & environmental sciences

For a reference copy of the document with all sections, see nature.com/documents/nr-reporting-summary-flat.pdf

Life sciences study design

All studies must disclose on these points even when the disclosure is negative.

Sample size	This is a cross-sectional omics study not intervention study, and sample size calculations are generally not performed.
Data exclusions	Individuals were excluded if they met one of the following criteria: diagnosed with acute or chronic inflammatory diseases, infectious diseases, chronic gastrointestinal disease, other severe organic lesions, or metabolic diseases, or received antibiotics, probiotics, prebiotics, or any other medical treatment within one month.
Replication	All attempts at replications were successful. We performed metagenomic and metabolomic analyses in the independent discovery and validation cohort, and key findings from the discovery cohort could be validated in the validation cohort. Moreover, the results of the stability of the characteristics of gut microbiota assessed by 16S rRNA gene sequencing were replicated by metagenomic sequencing.
Randomization	In the animal experiments, mice were randomly assigned to experimental groups, and animals assigned to different experimental conditions were run in parallel.
Blinding	All analyses were performed blinded to the identity and clinical characteristics of the participants. For animal studies, investigators were not blinded to allocation during experiments but blinded to outcome assessments.

Reporting for specific materials, systems and methods

We require information from authors about some types of materials, experimental systems and methods used in many studies. Here, indicate whether each material, system or method listed is relevant to your study. If you are not sure if a list item applies to your research, read the appropriate section before selecting a response.

Materials & experimental systems

n/a	Involved in the study	
[ ]	[x]	Antibodies
[x]	[ ]	Eukaryotic cell lines
[x]	[ ]	Palaeontology and archaeology
[ ]	[x]	Animals and other organisms
[ ]	[x]	Human research participants
[x]	[ ]	Clinical data
[x]	[ ]	Dual use research of concern

Methods

n/a	Involved in the study	
[x]	[ ]	ChIP-seq
[x]	[ ]	Flow cytometry
[x]	[ ]	MRI-based neuroimaging

Antibodies

Antibodies used	Western blot analysis:<br>Primary antibodies: TLR4 (Proteintech Group, Rosemont, IL, USA; 19811-1-AP), MyD88 (Proteintech Group, 23230-1-AP), NF-κB p65 (Immunoway, Plano, TX, USA; YM3111), phospho-NF-κB p65 (Immunoway, YP0847), and β-actin (Immunoway, YM3028). Dilutions for primary antibodies and secondary antibodies were 1:1000 and 1:10000 respectively.<br><br>Immunohistochemistry:<br>anti-insulin primary antibody (Servicebio, GB13121, 1:300), goat anti-mouse HRP conjugated secondary antibody (Servicebio, GB23301, 1:200)
Validation	Validation and relevent references of primary antibody are provided on the manufacturers' website:<br>TLR4 (https://www.ptgcn.com/products/TLR4-Antibody-19811-1-AP.htm#publications),<br>MyD88 (https://www.ptgcn.com/products/MYD88-Antibody-23230-1-AP.htm),<br>NF-κB p65 (hhttp://www.immunoway.com/Home/22/YM3111),<br>phospho-NF-κB p65 (http://www.immunoway.com/Home/22/YP0847),<br>β-actin (http://www.immunoway.com/Home/22/YM3028),<br>Insulin (https://www.servicebio.cn/goodsdetail?id=646),

	HRP-conjugated goat anti-mouse IgG (https://www.servicebio.cn/goodsdetail?id=264).

Animals and other organisms

Policy information about studies involving animals; ARRIVE guidelines recommended for reporting animal research

Laboratory animals	C57/BL6 male mice (6 weeks old) were purchased from Beijing Wei Tong Li Hua Laboratory Animal Technology Co., Ltd., Beijing, China. C57/BL6 mice were housed under specific pathogen-free conditions. Germ-free mice (8 weeks old, C3H/Orl male mice) were purchased from Shanghai Slack Laboratory Animal Co., Ltd and were housed at the gnotobiotic facility under strict germ-free conditions. Mice were kept under standard laboratory conditions (12:12 light/dark cycle, 22°C; 55-60% humidity; food and water ad libitum).
Wild animals	The study did not involve wild animals.
Field-collected samples	The study did not involve samples collected from the filed.
Ethics oversight	The study was approved by the Institutional Review Board and Ethics Committee of Children's Hospital of Fudan University ([2021] 181).

Note that full information on the approval of the study protocol must also be provided in the manuscript.

Human research participants

Policy information about studies involving human research participants

Population characteristics	Through a strict pathological diagnosis and exclusion process, new-onset T1D and NC subjects were enrolled in discovery and validation cohort respectively. The two groups were balanced for age, sex, delivery and feeding manner. Detailed information was shown in the Table 1.
Recruitment	This was a multi-center and cross-sectional study, centering on the Children's Hospital of Fudan University with other top-level tertiary hospitals in nine regions in China. A total of 158 subjects in the discovery cohort and 65 in the validation cohort were initially recruited from nine regions in China from north to south including Harbin, Changchun, Taiyuan, Jinan, Zhengzhou, Suzhou, Shanghai, Nanchang and Fuzhou between January 2018 and July 2019. The control subjects were recruited from individuals who visited the outpatient clinic for health status check-ups. Children with T1D were firstly diagnosed according to the American Diabetes Association diagnostic criteria. These newly diagnosed T1D children were divided into two subgroups: those with DKA and those without DKA. Finally, 77 non-diabetic controls and 64 children with new-onset pediatric T1D were included in the discovery set, with 60.94% of T1D children experiencing DKA (n=39) at T1D diagnosis. 29 NC children and 29 children with T1D were included in the validation set, with 58.62% of T1D children experiencing DKA (n=17). To minimize potential biases such as selection bias and confounding, our study was originally designed as a multi-center study a large sample size, and potential study subjects who met inclusion and exclusion criteria were entered into the study. And the NC and T1D group were matched for age, sex, delivery, and feeding mode.
Ethics oversight	Written informed consent was taken from all the study participants before sample collection and the study was approved by the Institutional Review Board and Ethics Committee of Children's Hospital of Fudan University ([2016]210 and [2019]210).

Note that full information on the approval of the study protocol must also be provided in the manuscript.
